# Ceramic Nanocomposites from Tailor-Made Preceramic Polymers

**DOI:** 10.3390/nano5020468

**Published:** 2015-04-01

**Authors:** Gabriela Mera, Markus Gallei, Samuel Bernard, Emanuel Ionescu

**Affiliations:** 1 Institut für Materialwissenschaft, Technische Universität Darmstadt, Jovanka-Bontschits-Strasse 2, D-64287 Darmstadt, Germany; E-Mail: mera@materials.tu-darmstadt.de; 2 Ernst-Berl-Institut für Technische und Makromolekulare Chemie, Technische Universität Darmstadt, Alarich-Weiss-Strasse 4, D-64287 Darmstadt, Germany; E-Mail: m.gallei@mc.tu-darmstadt.de; 3 Institut Européen des Membranes (UMR 5635-CNRS/ENSCM-UM2) CC 047-Place E. Bataillon, 34095 Montpellier Cedex 05, France; E-Mail: samuel.bernard@univ-montp2.fr; 4 Department Chemie, Institut für Anorganische Chemie, Universität zu Köln, Greinstrasse 6, D-50939 Köln, Germany

**Keywords:** ceramic nanocomposites, polymer-derived ceramic nanocomposites (PDC-NCs), preceramic polymers, metallopolymers, polymer-to-ceramic conversion

## Abstract

The present Review addresses current developments related to polymer-derived ceramic nanocomposites (PDC-NCs). Different classes of preceramic polymers are briefly introduced and their conversion into ceramic materials with adjustable phase compositions and microstructures is presented. Emphasis is set on discussing the intimate relationship between the chemistry and structural architecture of the precursor and the structural features and properties of the resulting ceramic nanocomposites. Various structural and functional properties of silicon-containing ceramic nanocomposites as well as different preparative strategies to achieve nano-scaled PDC-NC-based ordered structures are highlighted, based on selected ceramic nanocomposite systems. Furthermore, prospective applications of the PDC-NCs such as high-temperature stable materials for thermal protection systems, membranes for hot gas separation purposes, materials for heterogeneous catalysis, nano-confinement materials for hydrogen storage applications as well as anode materials for secondary ion batteries are introduced and discussed in detail.

## 1. Introduction

Multifunctional materials are capable of providing two or more primary functions either in a simultaneous manner or sequentially. For instance, one defines the category of multifunctional structural materials, which exhibit additional functions beyond their basic mechanical strength or stiffness (which are typical attributes of structural materials). Thus, they can be designed to possess incorporated electrical, magnetic, optical, sensing, power generative or other functionalities, which work in a synergistic manner [1]. The basic motivation for the development of multifunctional materials relies on their ability to address several mission objectives with only one structure—thus, they are capable of adapting on purpose their performance and response depending on the specific target application. Multi-mission objectives can be addressed simultaneously or consecutively by using multiple structures. However, due to ever-growing number of needed functions, the number of the individual objectives of the respective structures is becoming prohibitive, *i.e.*, the limiting factor concerning the design of suitable functional materials and devices [2]. Also specific aspects concerning the storage, maintenance, interactions, transport of the individual components might become critical.

Consequently, multifunctional materials represent the ultimate solution to address and provide multiple functions with one sole structure. They are usually (nano)composites or (nano)hybrids of several distinct (Gibbsian) phases (*i.e.*, phases with specific, individual chemical composition and physical state), each of them providing a different but essential function. Optimized design of multifunctional materials allows for having no or less “non-function” volume and thus provide significant advantages as compared to the traditional multicomponent “brass-board” systems: They are more weight and volume efficient, exhibit high flexibility with respect to their function(s) and performance, as well as are potentially less prone to maintenance issues [3].

Within the present Review, different classes of ceramic nanocomposite materials prepared from tailored preceramic polymers will be highlighted and discussed. One main emphasis of the critical discussion in the present Review will relate to the intimate relationship between the chemistry and macromolecular architecture of the preceramic polymers and the phase composition, microstructure and properties of the resulting ceramic nanocomposites. Following a Section related to the preparative access to ceramic nanocomposites from suitable preceramic polymers, a detailed description of various structural and functional properties followed by selected examples of prospective applications will be given.

## 2. Preceramic Polymers

### 2.1. Silicon-Containing Preceramic Polymers

The current research is moving forward to establishing novel preparative strategies to produce tailor-made silicon-based preceramic polymers [4] as precursors for polymer-derived ceramics (PDCs) [5,6]. Within this context, studies concerning the intimate relationships between their molecular architecture and the microstructure and properties of the ceramic materials resulting there from are of crucial importance [5,7,8,9] The thermolysis of Si-based preceramic polymers under specific atmosphere and heat treatment conditions represents a straight-forward and inexpensive additive-free process [5,10] which allows to control and adjust the phase composition and the microstructure and thus the materials properties of ceramic components. Consequently, there is a stringent need in developing designed preceramic polymers, with tailored molecular architecture, physico-chemical properties and suitable ceramization behavior.

**Figure 1:**
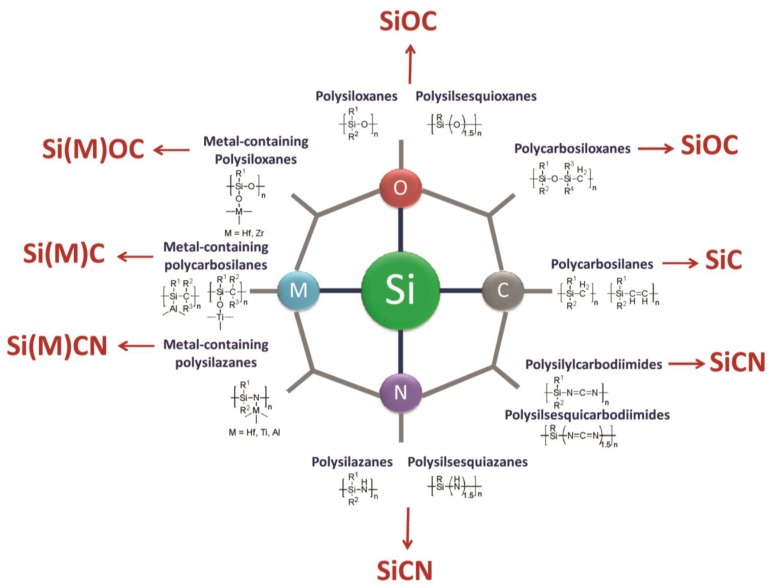
Typical classes of organosilicon polymers used as precursors for ceramics (reprinted with permission from Wiley) [4].

The general classes of silicon-based polymers used as precursors for ceramics, *i.e.*, polysilanes, polycarbosilanes, polysiloxanes, polysilazanes and polysilylcarbodiimides are shown in Figure 1 and will be briefly discussed in the following. Metal-containing polysiloxanes, polysilazanes and polycarbosilanes will be highlighted and discussed in Section 2.2 of the present paper.

Following requirements should be fulfilled by preceramic polymers in order to be suitable for the production of polymer-derived ceramics: (i) the polymers should possess a sufficiently high molecular weight in order to avoid volatilization of low molecular components; (ii) they should have appropriate rheological properties and solubility for the shaping process and (iii) latent reactivity (presence of reactive, functional groups) for the curing and cross-linking step.

The synthesis of ceramic materials starting from preceramic polymers has several advantages as compared to other preparative methods: (i) pure starting compounds (precursors); (ii) possibility to modify the molecular structure of the precursors by a variety of chemical reactions; (iii) application of shaping technologies well known from plastic forming; (iv) easy machining of the green body; (v) relatively low synthesis temperatures in comparison with classical ceramic powder processing technologies; (vi) preparative access to ceramic systems which cannot be produced by other methods (*i.e.*, ternary and multinary systems such as SiOC, SiCN, *etc.*).

*Polysilanes* (also named polysilylenes) [11,12,13,14] represent a class of polymeric materials composed of an one-dimensional silicon chain and organic groups attached at silicon and exhibit rather unique optoelectronic and photochemical properties related to the extensive delocalization of σ electrons along silicon backbone (σ conjugation). Polysilanes are used as photoresists in microlithography, as well as they found applications as photoconducting polymers, third-order nonlinear optical materials and as valuable precursors for the synthesis of additive-free silicon carbide [11,15].

Polysilanes are typically unstable in air and moisture and suffer from degradation when exposed to UV light. Due to their insolubility, non-meltability and intractability, their processability is rather challenging. Their properties are significantly depending on their molecular weight, as well as on the nature of the organic groups attached at silicon and the conformation (branching) of the polymer chain.

The synthesis of polysilanes is mainly done by using the Wurtz-Fittig reductive coupling reaction of chlorosilanes with sodium or lithium in boiling toluene, benzene or tetrahydrofurane [16,17]. Soluble homo- and copolymers, mainly methyl-containing polysilanes, were reported in the late 70’s [18,19,20]. Despite the fact that this synthesis method for polysilanes is rather old and yields polymers with structures, molecular weight and polydispersities difficult to control, it is still the most common method of choice for the synthesis of polysilanes. Moreover, the Wurtz-Fittig reaction is highly sensitive on the nature and dispersion of the used alkali metals (Na, Li, K, Na/K alloy), on the solvents and additives, on reaction temperature, *etc.* [21,22,23,24,25].

An important salt-free, high-yield synthesis method for high-purity polysilanes is the dehydrocoupling reaction of hydridosilane in the presence of catalysts such as η^2^-alkynyl titanocene or -zirconocene [26]. Also zirconocene and hafnocene hydride have been reported as suitable catalyst for the dehydrocoupling polymerization from hydridosilanes [27].

Other suitable methods used for the synthesis of polysilanes are the anionic polymerization of masked disilenes [28] ring-opening polymerization of strained cyclosilanes [29] and recently, reduction reaction of chlorosilanes with Mg in the presence of LiCl and a Lewis acid under mild condition [30].

**Figure 2:**
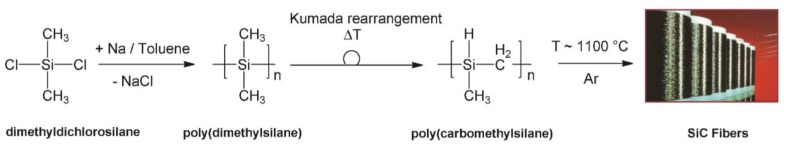
Yajima process for the synthesis of silicon carbide (SiC) ceramic fibers (reprinted with permission from Wiley) [4].

Among polysilanes, polydimethylsilane represents an important precursor for silicon carbide fibers as reported for the first time by Yajima *et al.* in 1975 [31]. Thermal treatment of polydimethylsilane at *ca.* 400 °C leads to the formation of a soluble and processable polycarbosilane (*i.e.*, polymethylsilylenemethylene), which can be easily spun into fibers or casted. This process, known as Kumada rearrangement or Kumada reaction, consists in a radical-induced methylene migration from one of the methyl substituents attached at Si into the polymer chain (Figure 2) [32]. The fibers obtained from polycarbomethylsilane are subsequently cured (thermal treatment in air or alternatively e-beam curing) and pyrolyzed at ~1100–1300 °C in argon atmosphere to yield SiC fibers known under the commercial name of Nicalon™ or High Nicalon™.

*Polycarbosilanes* having the general formula–[R^1^R^2^Si-C(R^3^)(R^4^)]*_n_*–(R^1^, R^2^, R^3^, R^4^ being H or organic groups) can be synthesized by several methods, such as the Kumada rearrangement of polysilanes [32], Grignard polycondensation reactions of chlorosilanes [33], ring-opening polymerization of 1,3-disilacyclobutanes catalyzed by Pt-containing complexes [34,35], dehydrocoupling reaction of trimethylsilane or hydrosilylation of vinylhydridosilanes.

Unsaturated polycarbosilanes represent a special class of hybrid polymers with silicon being bonded to π-conjugated building blocks, such as phenylene, ethenylene, or diethylene [36,37,38,39,40]. The synthesis of these materials has been achieved by using coupling reactions [38,41] thermal cyclopolymerization [42,43] and a variety of ring-opening polymerization reactions including anionic [44,45,46,47], thermolytic and catalytic coordination techniques [48], each of them with some limitations. A novel approach for the synthesis of unsaturated polycarbosilanes involves the catalytic acyclic diene metathesis (ADMET) reaction of unsaturated oligosilanes in the presence of a ruthenium carbene complex RuCl_2_(PCy_3_)_2_(=CHPh) (Grubbs catalyst) [49].

*Polysiloxanes* show excellent chemical and physical properties and have been extensively used as suitable single-source precursors for the synthesis of silicon oxycarbide (SiOC) ceramic materials via pyrolysis in inert or reactive atmosphere [5,7]. They exhibit outstanding thermo-mechanical properties owing to the combination of relatively unique features such as a pronounced elasticity at low temperatures, or high stability at elevated temperatures and in oxidative environments. The low temperature elasticity of polysiloxanes is manifested in some of the lowest glass transition temperatures known to polymers, moreover in low crystalline melting points, fast crystallization processes, specific liquid crystalline behavior and small viscosity-temperature coefficients. These properties rely on the pronounced polymer segmental chain mobility in polysiloxanes, which is correlated to the inherent chain flexibility (large Si–O–Si angles from 140° to 180°) and relatively weak intra- and intermolecular interactions [50].

The high temperature stability of polysiloxanes concerning decomposition is also related to the inherent strength of the siloxane bond as well as to the pronounced flexibility of –(Si-O-Si-O)*_x_*– segments. The partial ionic and double bond characters of the Si–O bond lead to its exceptional strength, since both effects increase the binding force between the participating silicon and oxygen atoms. This relies on the unique d_π_–p_π_ bond between Si and O resulting in an Si–O bond dissociation energy of about 108 kcal·mol^−1^, which is considerably higher than those of single bonds such as C–C (82.6 kcal·mol^−1^), C–O (85.2 kcal·mol^−1^) or even C_arom_–C bonds (97.6 kcal·mol^−1^) [50]. Thus, the Si–O bond withstands exposure to higher temperatures than the bonds normally found in organic polymers, leading to a significantly higher thermal stability of polysiloxanes than that of their organic (C–C) counterparts.

Polysiloxanes are synthesized starting from functionalized silanes, *i.e.*, R*_x_*SiX_4−*x*_ (with *x* = Cl, OR, OC(=O)R or NR_2_ and R = alkyl, aryl groups). One of the most used functionalized silanes for the industrial synthesis of polysiloxanes is dimethyl dichloro silane, which is obtained via the so-called Direct Process, involving the copper-catalyzed reaction of gaseous chloromethane with silicon in fluidized- or stirred-bed reactors at temperatures of *ca.* 250–300 °C (the Müller-Rochow process). After a subsequent destillative purification of dimethyl dichloro silane from other Me*_x_*SiCl_4−*x*_ (*x* = 1, 3, 4) side products, the general route to obtain polysiloxanes consists mainly of two steps: (a) hydrolysis of the dichloro dimethylsilane, which leads to the formation of a mixture of linear and cyclic oligosiloxanes; and (b) polycondensation of hydroxyl-functionalized short-chain polysiloxanes or ring-opening polymerization processes of the cyclic oligomers which lead to high molecular weight polysiloxanes [50].

In order to provide a high ceramic yield, polysiloxanes have to exhibit a high degree of cross-linking. This can be achieved by using suitable functional chain groups in linear polysiloxanes which allow for thermal or irradiation-assisted (e.g., UV light) curing and cross-linking or by using highly cross-linked polysiloxanes. Within this context it has been shown that polysilsesquioxanes, of general formula RSiO_1.5_ (with R being H or an organic group) are suitable for being used as preceramic polymers, since they exhibit a highly branched molecular architecture and consequently lead to high ceramic yields [5,7]. Cross-linked polysiloxanes or silicon resins can furthermore be synthesized through sol–gel processes via hydrolysis and condensation of hybrid silicon alkoxides. This type of precursors has been used since end of the 80s to synthesize silicon oxycarbide glasses [51,52]. They are modified silicon alkoxides of the general formula R*_x_*Si(OR')_4−*x*_ (R = alkyl, allyl, aryl; R' = methyl, ethyl), which upon gelation convert into silicone resins of the composition R*_x_*SiO_(4−*x*)/2_. As different hybrid silicon alkoxides can be used for co-hydrolysis and subsequent polycondensation, the sol–gel preparative access to silicone resins allows for controlling and tuning their compositions. Consequently, single-source precursors for stoichiometric silicon oxycarbide, as well as for Si–O–C materials showing excess of carbon or silicon can be prepared [53]. Moreover, this preparative technique allows for introducing additional elements within the preceramic network, e.g., Al, Ti, B, by using suitable metal alkoxides [6]. For instance, functionalized silanes such as Si(OR)*_x_*R'_4−*x*_ (R, R' = alkyl groups) are reacted with titanium isopropoxide [54], zirconium *n*-propoxide [55] or di-*tert*-amyloxy-vanadate [56] in order to obtain so‑called hybrid gels containing homogeneously dispersed transition metals within the gel backbone. The synthesis of hybrid materials comprising of siloxane-type precursors and metal alkoxides has been known since the late 80s. While at the beginning the focus of the investigation was set rather on elucidating the molecular structure and network architecture of the metal-modified sol–gel materials [56,57,58,59], later on studies related to their conversion into ceramic nano-composites became more and more numerous and attractive [54,60,61,62,63].

*Polysilazanes* are polymers containing silicon and nitrogen within their backbone and have been used during the last decades to synthesize amorphous silicon nitride and silicon carbonitride ceramics [64].

First attempts were made to synthesize silicon nitride by thermal conversion of Si[N(CH_2_CH_3_)_2_]_4_ in argon atmosphere. This process was expected to occur similarly to the formation of silica from silicon alkoxides. However, it leads to the formation of silicon carbonitride, SiCN [65]. Carbon-free polysilazanes were synthesized already in 1885 via ammonolysis of tetrachloro silane in liquid phase [66]. This process delivers silicon diimide, which converts upon thermal treatment at *ca.* 1000 °C in inert gas atmosphere, into amorphous silicon nitride and gaseous ammonia [67].

Perhydridopolysilazane (PHPS) represents also a carbon-free precursor for silicon nitride. The synthesis of PHPS is achieved via ammonolysis of dichloro silane (SiH_2_Cl_2_) in polar solvents. The product consists of low molecular linear and cyclic oligomers, which however cross-link rapidly upon hydrogen loss and lead to highly viscous (up to glassy) polysilazane [68]. Its thermal treatment at 1000 °C in nitrogen atmosphere leads to a mixture of α-Si_3_N_4_, β-Si_3_N_4_ and excess silicon. If the thermal treatment of the PHPS is performed in ammonia atmosphere, the formation of elemental silicon is suppressed. Due to the high reactivity of dichloro silane (which is highly flammable and moreover disproportionates into silane, SiH_4_ and tetrachloro silane, SiCl_4_), the process was modified by using H_2_SiCl_2_*(NC_5_H_5_)_2_ [69]. A further improvement of the process involves the co-ammonolysis of dichloro and trichloro silanes, leading to preceramic polymers which are able to be thermally converted into stoichiometric Si_3_N_4_ [70].

*Polycarbosilazanes* are usually prepared upon ammonolysis or aminolysis of halogeno-substituted organyl silanes, e.g., R*_x_*SiCl_4−*x*_. In a first substitution step a chlorine substituent is replaced by a –NH_2_ group; subsequently, condensation reactions occur, leading to the formation of Si–N–Si linkages. Depending on the number of the chlorine substituents as well as on the nature and size of the organic substituents R in R*_x_*SiCl_4−*x*_, different types of silazanes can be obtained, such as linear or cyclic, oligomers or highly cross-linked polymers. Starting from R_2_SiCl_2_, mixtures of cyclic oligomers and low molecular weight linear polymers are obtained, which can be further cross-linked, usually via thermal treatment. In the case of Si–H and N–H containing silazanes, cross-linking can be achieved by means of addition of bases in catalytic amounts (e.g., potassium hydride—KH) [71].

Polysilazanes can be chemically modified by reactions with transition metal alkoxides, as it has been shown for Al or group IV metal alkoxides (M = Ti, Zr, Hf). In different studies, hydrido- [72,73] or methyl-/vinyl-substituted polysilazanes [6,62,74,75] were reacted with group IV metal alkoxides. In the case of hydrido-polysilazane, the reaction with titanium *n*-butoxide takes place at the N–H groups upon formation of Si–Ti linkages. The reaction of HTT1800 with hafnium *n*-butoxide was shown to occur at both N–H and Si–H functional groups as confirmed by Raman and ^1^H‑NMR spectroscopy [74]. Polysilazanes can be modified also with non-oxidic organometallics. Recently, several studies concerning the chemical modification of PHPS as well as vinyl- and hydrido-substituted polysilazanes with Ti [72,73,76,77], Zr and Hf [78,79] amido complexes were reported. The obtained metal-containing single-source precursors were shown to be highly compliant and to provide access to different ultrahigh-temperature stable ceramic nanocomposites (such as MN/Si_3_N_4_, MN/SiCN, MCN/SiCN, *etc.*, with M = Ti, Zr, Hf), depending on the conditions used for the ceramization.

*Polysilylcarbodiimides* are valuable precursors for the synthesis of SiCN-based ceramics [5,7,80]. The synthesis of polysilylcarbodiimides was firstly reported by Ebsworth, Wannagat and Birkofer [81,82,83,84]. They have been shown to be useful as stabilizing agents for polyurethanes and polyvinylchloride, as insulator coatings, high temperature stable pigments [85] and as irradiation-resistant sealing materials [86]. Moreover, polysilylcarbodiimides have been used for the synthesis of organic cyanamides, carbodiimides and heterocycles [87]. Polysilylcarbodiimides are generally air and moisture sensitive [88]. Upon insertion of bulky aromatic substituents at silicon, their air sensitivity significantly decreases [80]. Carbon-rich poly(phenylsilylcarbodiimide) derivatives, namely –[PhRSi-NCN]*_n_*–, (R = H, methyl, vinyl, phenyl) were synthesized by the reaction of phenyl-containing dichlorosilanes with bis(trimethylsilylcarbodiimide) in the presence of pyridine as catalyst [80,89]. These polymers show an increased stability against air and moisture and were shown to be suitable precursors for carbon-rich nanostructured SiCN ceramics [5,7,80,89,90,91,92,93].

Synthesis of polysilylcarbodiimides can be performed upon reacting of di-, tri- and tetrachlorosilanes with silvercyanamide, as reported by Pump and Rochow in 1964 [94], as well as via trans-silylation of bis(trimethylsilylcarbodiimide) with chlorosilanes as shown in 1968 by Klebe and Murray [85]. Alternatively, polysilylcarbodiimides can be obtained by the polycondensation reaction of cyanamide with chlorosilanes [88]. The most appropriate method for the synthesis of polysilylcarbodiimides is the pyridine-catalyzed polycondensation reaction of chlorosilanes (di, tri- and tetra- chlorosilanes) with bis(trimethylsilylcarbodiimide) [5,7,80,88,91,95,96,97]. The scientific interest on polysilylcarbodiimides increased as Riedel *et al.* [88,95,98,99,100,101] reported in the 90s on their thermal transformation to SiCN ceramics.

Starting from dichloro silanes, cyclic or linear polymers can be obtained. Trichloro silanes yield by the reaction with bis(trimethylsilylcarbodiimide) a class of branched polymers, namely polysilsesquicarbodiimides. Depending on the branching of the chain, different microstructures and thermal stabilities were found in their derived ceramics [91]. Interestingly is the reaction of tetrachloro silane with bis(trimethylsilylcarbodiimide) [100]. The decomposition of this highly branched polymer yield up the first ternary crystalline phases in the Si–C–N system, namely SiC_2_N_4_ and Si_2_CN_4_ [100].

**Figure 3:**
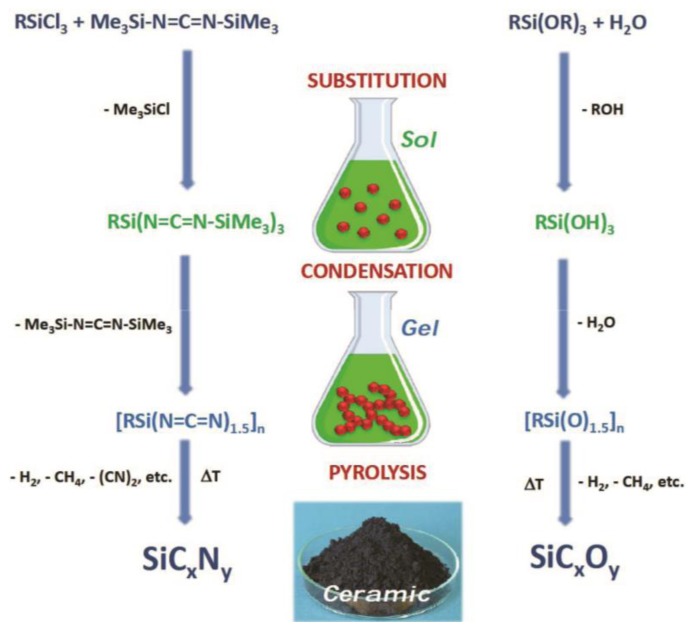
Comparison between the non-oxide sol–gel process for the synthesis of polysilylcarbodiimides and the oxide sol–gel process for the synthesis of polysiloxanes.

Due to the pseudochalcogen character of the (NCN)^2−^ anion,[102] polysilylcarbodiimides were shown to exhibit similar properties to those of polysiloxanes [5,7]. Indeed, the non-oxidic sol–gel process of trichlorosilanes with bis(trimethylsilylcarbodiimide) follows the same steps as the sol–gel process of trialkoxysilane with water (Figure 3) [88].

### 2.2. Single-Source Precursors Based on Metallopolymers

Compared to the previous section which comprised feasible tailor-made ceramics featuring high ceramic yields based on the conversion of, e.g., polysiloxanes, polycarbosilanes and polysilazanes (*cf.* Section 2.1), in this section metallopolymers will be highlighted as suitable ceramic precursors. Recently, metal-containing polymers attracted enormous attention due to their promising combination of redox, mechanical, semi-conductive, photo-physical, optoelectronic, magnetic and catalytic properties [103,104,105,106,107,108,109]. Such kinds of polymers can either feature (i) a metal center as integral part of the polymer main chain or (ii) the metal is laterally attached to the polymer chain. As a further structural distinction of metallopolymers—which definitely affect the ceramic yield of the final functional ceramic—these polymers can be distinguished by a linear, dendritic or hyperbranched structure. The preparation of nano- and micro-structured ceramics based on block copolymers and colloidal architectures will be discussed in the ensuing Section 4.3. Selected examples for the conversion of metallopolymers into ceramics will be addressed within this section. They are mainly synthesized to produce carbide nanocomposites.

A soluble poly-yne carbosilane copolymer with sandwiched zirconium moieties was synthesized as a preceramic metallopolymer for the preparation of ZrC/SiC/C ternary composite ceramics. Thermal treatment of the precursor polymer at a temperature of 1400 °C revealed a ceramic yield of over 52% of a highly crystalline material [110]. Another strategy for the preparation of nano-sized ZrC composites with a carbon fiber reinforced carbon matrix was reported by Tao *et al.* [111]. The authors took advantage of an air-stable and processable zirconium-precursor, polyzirconosaal, for infiltration followed by pyrolysis. Here, the ceramic yield for the final composite yielded 58% of a ZrC-C/C composite after thermal treatment. Very recently, Wu *et al.* [112] described a method for the preparation of zirconium-based precursor polymers starting from zirconium tetrachloride. While zirconium oxide was initially formed from these polymers at a rather moderate temperature of 1200 °C, crystalline ZrC particles exhibiting face-centered cubic lattice structures (50 to 100 nm) were accessible by subsequent thermal treatment at 1400 °C. ZrC composites have also been prepared by a polymer-analogous route, *i.e.*, by post functionalization of a reactive polycarbosilane [113]. As described in the previous section, hydrosilylation is a powerful method for this purpose. Wang *et al.* [113] used a zirconocene derivative tethered at a polycarbosilane backbone for the preparation of ZrC/SiC composites after thermal treatment. The final materials were accessible with a remarkable ceramic yield of 78%.

An interesting approach for the formation of mixed lanthanide coordination polymers for the preparation of rare earth oxides was reported by Demars and coworkers [114]. Different shapes of investigated oxides have been found simply by varying the preparation methods of the metal-containing polymeric precursor material. Well-defined cylindrical or spherical micro-morphologies could be adjusted by changing the used solvents (water or tetrahydrofurane, THF) which retained in the final oxides after thermal treatment [114].

**Figure 4:**
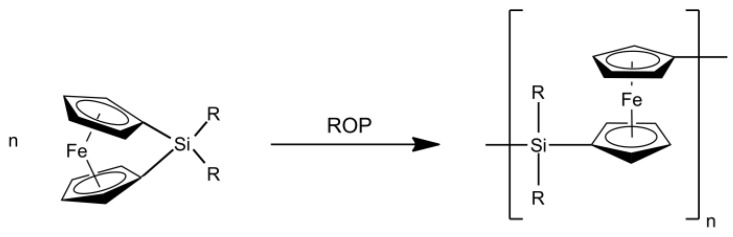
Ring opening polymerization (ROP) of *ansa*-silaferrocenophanes yielding poly(ferrocenylsilane) (R = alkyl).

The vast majority of reports deal with metallocene-containing polymers, *i.e.*, metal centers sandwiched between cyclic hydrocarbon moieties, as recent synthetic pathways led to stable and well characterized functional materials [115]. Noteworthy, such metallopolymers have been used for the preparation of block copolymers featuring the intrinsic capability of self-assembling into well-ordered nano-scaled structures. Within the field of metallocene-based polymers, especially ferrocene-containing polymers were found to be powerful precursor materials for the preparation of iron-based ceramics with remarkably high ceramic yields. The most prominent example in the field of ferrocene-containing polymers for the conversion into functional ceramic materials was reported by the group of Manners. By ring-opening polymerization (ROP) of ring-strained *ansa*-silaferrocenophanes, polyferrocenylsilanes (PFS) for the preparation of well-defined nanostructured ceramics could be obtained (Figure 4) [116,117,118].

As shown on Figure 4, PFS belong to a class of metallopolymers that consist of alternating ferrocene and organosilane units in the polymeric backbone. An important feature of these polymers is their glass transition temperature which reflects their conformational flexibility. For instance, polyferrocenyldimethylsilane is a film-forming thermoplastic and can be melt-processed into various shapes such as nanofibers by electrospinning [119]. Thus, high-molecular weight polyferrocenylsilanes with linear, cyclic, or hyperbranched architecture and their block copolymers have shown potential in the preparation of shaped magnetic ceramics [120,121,122,123,124,125] and the self-assembly into well-defined hybrid architectures such as micelles [126], spheres [118,127], cylinders [128] and one-dimensional nanostructures [129,130]. Interestingly, PFS-derived ceramics possess tunable magnetic properties between the ferromagnetic and the superparamagnetic state, which can be achieved upon controlling the pyrolysis conditions of PFS.

Clendenning *et al.* [116] reported the usability of PFS films for the preparation of ferromagnetic ceramic films by reactive ion etching using a plasma. For this purpose, the PFS precursor was additionally functionalized with cobalt clusters at the silicon moiety. Ordered arrays of ferromagnetic ceramics could thus be obtained. As another example, PFS-based metallopolymers have been doped with palladium(II) acetylacetonate in order to produce soft processable polymer films [131]. Pyrolysis of this tailor-made preceramic film at 600–900 °C led to ferromagnetic ceramics, while higher applied temperature (1000 °C) led to the formation of FePd alloys [131]. Häußler and coworkers studied hyperbranched poly(ferrocenylene)s as feasible metallopolymers for a pyrolytic transformation into magnetic ceramics [132]. The hyperbranched polymeric framework was advantageous regarding the ceramic yield of magnetic iron nano-clusters featuring high magnetic susceptibilities. Moreover, pyrolysis in an argon atmosphere at 1200 °C led to the formation of iron silicide. The ferrocene-containing polymer poly(2-(methacryloyloxy)ethyl ferrocenecarboxylate) (PFcMA) which is accessible by using controlled polymerization or emulsion polymerization protocols seems to be an excellent candidate for the preparation of iron-based ceramics [133,134,135,136]. Mazurowski and coworkers succeeded to convert dense PFcMA brushes attached on organic particles into spherical iron oxides after thermal treatment [137]. Within this work, the chain length of the preceramic polymer as well as the polymer grafting density at the particle surface could be adjusted in a wide range. Recently, Yu *et al.* [138] used the hydrosilylation reaction of vinyl ferrocene with allylhydridopolycarbosilane (AHPCS) to synthesize a processable hyperbranched polyferrocenylcarbosilane. The polymer led to SiC/C/Fe nanocomposites with particular magnetic properties depending on the iron content in the polymer and on the pyrolysis conditions.

By combining the emulsion polymerization protocols of FcMA and the Stöber process, preceramic copolymers based on PFcMA and poly(methyl methacrylate) (PMMA) led to uniform ferrocene-containing particles of adjustable diameters in the range of (100–500 nm) [133]. The core/shell architectures featuring the ferrocene moieties as integral part of the particle shell were proven useful as single-source magnetic ceramic precursors. After thermal treatment, nanorattle-type ceramic architectures featuring a magnetic iron oxide core could be prepared with potential applications in fields of sensing and stimuli-responsive nano-photonics [133].

The pyrolysis of ferrocene-containing phosphonium polyelectrolytes as feasible preceramic polymers yielded iron-rich nanoparticles with carbon-, phosphorus-, and oxygen-rich phases in good ceramic yields (46%) as recently reported by the group of Gilroy [139]. Moreover, this strategy was shown to be feasible to adjust the composition of the final advanced ceramic composite material. Similarly to polycarbosilanes (PCS), PFS can be modified with other elements. Indeed, it is well known that incorporation of boron at atomic scale in polycarbosilane allows improving silicon carbide (SiC) sintering and SiC crystallization rate [140]. When boron is incorporated into PFS to form ferrocenylboranes [141,142,143,144], ferrocenylborane polymers [145,146,147,148,149] and ferrocene-containing poly(boro)-carbosilanes [150], multifunctional ceramics with tailorable magnetic properties and high-temperature resistance can be achieved under suitable pyrolysis conditions.

**Figure 5:**
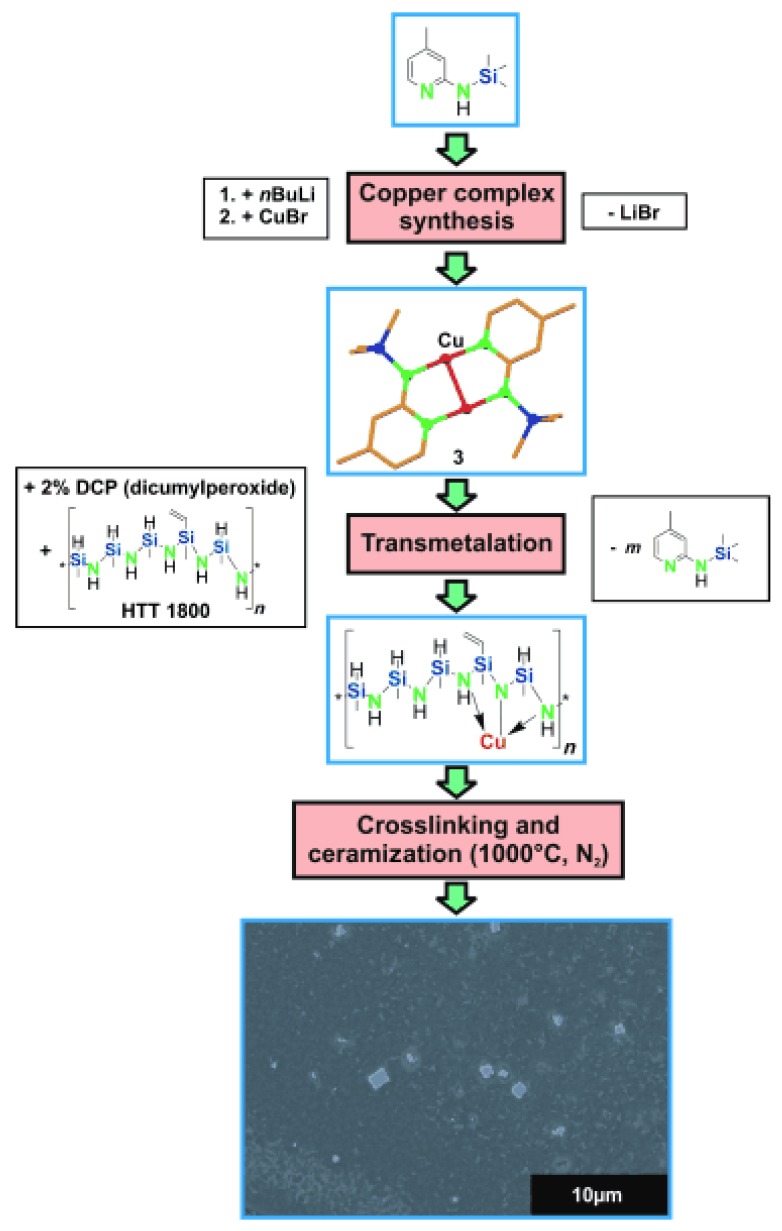
Modification of HTT1800 with an aminopyridinato copper complex leading to Cu-containing SiCN ceramics by pyrolysis to 1000 °C under nitrogen (with permission from Wiley) [152].

Previous examples described the modification of polycarbosilanes with ferrocene as pioneered by Manners. Final polymer-derived nanocomposites exhibit controlled magnetic properties (See Section 5.2). However, one of the advantages of polycarbosilanes and polycarbosilazanes is the availability of reactive groups within their structure such as Si–H, N–H, Si-vinyl, *etc.*, which allow their reaction with coordination compounds and thus the incorporation of late transition metals within the polymeric architecture. The resulting metal-containing polymers were shown to mainly to produce nanocomposites which exhibit promising catalytic properties (See Section 5.3) [151,152,153,154,155,156]. During the reaction, covalent bonds between the metal ions and the polycarbosilazanes can be established by the reaction with coordination compounds. During these metal transfer the ligand of the coordination compound is released and after cross-linking may become a part of the preceramic polymer. The synthesis is directed to produce metal-ceramic nanocomposites after pyrolysis during which *in situ* controlled growth of metal occurs in the matrix. As an illustration, a commercially available polycarbosilazanes, *i.e.*, HTT1800, was modified by aminopyridinato metal complexes. The aminopyridinato copper complex [Cu_2_(Ap^TMS^)_2_] (Ap^TMS^H = (4-methylpyridin-2-yl)trimethylsilanylamine) reacted with HTT1800 via transmetalation, *i.e.*, aminopyridine elimination (Figure 5) [152].

This reaction was investigated by ^1^H and ^13^C NMR spectroscopy. The driving force of this reaction was considered to be the low coordination number of copper in [Cu_2_-(Ap^TMS^)_2_] leading to the establishment of covalent bondings between the copper atoms and the polycarbosilazane. Crosslinking of the copper-modified polycarbosilazane and subsequent pyrolysis led to the copper-containing SiCN ceramics. Using an amido Nickel complex ([Ni(Ap^TMS^)_2_]_2_) to react with HTT1800 according to various and controlled Si:Ni ratios in THF was reported to generate a solid and dark polymer. The amido Nickel complex catalyzed the cross-linking of HTT1800 via hydrosilylation at room temperature. After pyrolysis to 600 °C under an inert atmosphere, Ni particles located near the external surface of the SiCN ceramic as well as within the internal voids were obtained [153]. The nanocomposites demonstrated catalytic activity for hydrogenation reactions (See Section 5.3). Recently, by changing the nature of the preceramic polymer and using a commercial allylhydridopolycarbosilane (AHPCS), Ni-containing SiC ceramics could be synthesized through the self-assembly of AHPCS-*block*-polyethylene (PE) [155]. The block copolymer was synthesized through Ni complex-catalysed dehydrocoupling of Si–H in AHPCS with O–H in the hydroxy-terminated PE. The added nickel complex ([Ni(Ap^TMS^)_2_]_2_ also catalyzed the cross-linking of the AHPCS block, for example, through dehydrocoupling reactions of the Si–H bonds. By changing the nature of the metal-containing precursor, *i.e.*, trans-[bis(2-aminoetanol-*N*,O)diacetato-nickel(II)], HTT1800 was chemically modified through (i) reaction between the OH groups in the complex and the Si centers of HTT1800; (ii) hydrosilylation reactions resulting in the formation of carbosilane bonds; and (iii) reduction of Ni^2+^ and *in-situ* formation of Ni nanoparticles in the polymer matrix. After pyrolysis at 700 °C, nanoporous silicon oxycarbonitride ceramics modified by Ni nanoparticles with a BET surface area of 215 m^2^·g^−1^ were obtained [156]. Kamperman *et al.* [151] applied micromolding and two-component colloidal self-assembly with cooperative assembly of a five component precursor system (solvent, amphiphilic block copolymer (poly(isoprene-*block*-dimethylaminoethyl methacrylate) (PI-*b*-PDMAEMA)), radical initiator (Dicumyl peroxide), commercial poly(ureamethylvinyl)silazane (PUMVS)) and the coordination compound [(COD)PtMe_2_] (COD = 1,5-cyclooctadiene) platinum catalyst precursors) to obtain the Pt@SiCN materials after heat treatment to 1000 °C. The authors assumed that platinum segregated in the PDMAEMA domains, as the allyl groups of the PUMVS can efficiently add to Pt in a similar fashion to the double bond coordination of Pt with 1,5-cyclooctadiene in the precursor molecule.

## 3. Polymer-to-Ceramic Transformation

**Figure 6:**
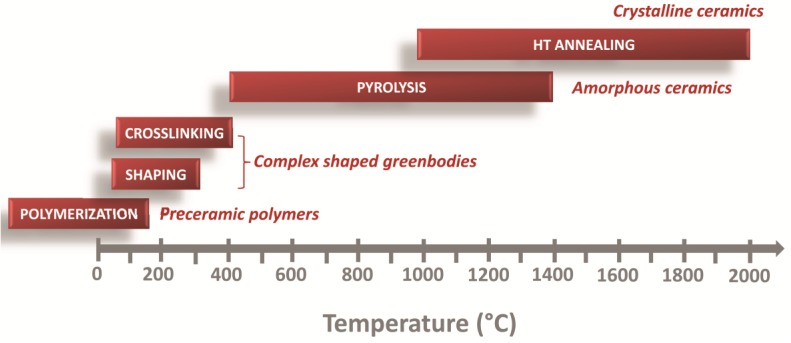
Polymer-to-ceramic transformation of preceramic polymers [6].

Controlled thermal decomposition of silicon-based polymers provides nano-structured ceramics with nanostructures strongly influenced by the chemistry and architecture of the precursors, their processing route and the parameters used for their pyrolysis (heating rate, reactive or inert atmosphere and dwelling time). Depending on the temperature, the preceramic polymers suffer different processes during their transformation to ceramics. After polymerization, shaping and cross-linking of the polymer can be easily done at moderate temperatures to obtain complex-shaped green-bodies which can retain their shape upon pyrolysis up to 1400 °C and also during high-temperature annealing up to 2000 °C. All these steps are defined by different chemical processes as discussed in the following (Figure 6).

The characterization of the individual polymer-to-ceramic conversion steps is usually done by combining several spectroscopic techniques such as multinuclear solid-state NMR, Raman, FTIR and XPS spectroscopy, with diffraction (X-ray diffraction, SAXS, electron diffraction) and microscopic techniques (SEM, TEM), as well as with elemental analysis.

### 3.1. Cross-Linking

During cross-linking the polymeric precursors are converted into organic/inorganic materials at low temperatures (up to 400–500 °C). This transformation prevents the loss of low molecular weight components of the polymer precursors as well as fragmentation processes during the pyrolysis process, and consequently leads to high ceramic yields. Furthermore, the cross-linking process allows for generating infusible materials (thermosets) which retain their shape during pyrolysis.

Cross-linking of polycarbosilanes can be achieved by thermal curing in air atmosphere or e-beam curing [157,158,159]. Cross-linking of polycarbosilanes in the presence of oxygen has been found to occur via radical mechanisms: oxidation of Si–H and Si–CH_3_ bonds occurs with the formation of Si–OH, Si–O–Si and C=O groups, as revealed by IR spectroscopy [160,161], XPS [162] and solid-state ^29^Si MAS NMR investigations [163]. Oxidative cross-linking of polycarbosilanes leads to SiC materials with oxygen contents of 10%–12%.

Cross-linking of polycarbosilanes in absence of oxygen involves reactions of Si–H bonds with Si–CH_3_ groups leading to Si–CH_2_–Si linkages as supported by IR spectroscopy [164] and solid-state ^29^Si MAS NMR [165] studies. Interestingly, no Si–Si bond formation occurs. Silicon carbide materials synthesized from e-beam cross-linked polycarbosilanes show very low oxygen content (0.2%–0.3%). This consequently leads to a strong improvement of their thermal stability and mechanical properties if compared to oxygen-rich SiC ceramics [10]. Cross-linking of polysiloxanes is achieved via condensation, transition metal catalyzed addition or free radical initiation techniques. In polymers containing methyl or vinyl groups cross-linking can be performed thermally by using peroxides [166].

The hydrosilylation reaction is an effective way to obtain infusible materials which are resistant toward water and elevated temperatures [167,168].

For polysiloxanes having functional groups in the structure, e.g., hydroxy or alkoxy groups, the cross-linking process occurs upon condensation of the silanol groups with *in situ* water release and subsequent hydrolysis reactions of the alkoxy substituents. Using appropriate catalysts, e.g., tetrakis(pentafluorophenyl)borate in the case of a polysiloxanol or [bis(2-ethylhexanoate)tin] in the case of poly(methoxymethylsiloxane) or poly(methylsiloxane), these reactions take place at room temperature [169,170].

Polysilazanes can be cross-linked either thermally and/or using reactive atmosphere such as ammonia and chemical reagents, such as catalysts or peroxides [64]. There are four major processes which can occur during the thermal cross-linking processes of polysilazanes: transamination, dehydrocoupling (between Si–H and N–H resp. Si–H and Si–H groups), vinyl polymerization and hydrosilylation.

Hydrosilylation reactions occur in (poly)silazanes which contain Si–H and vinyl substituents. This is a fast process which occurs at relatively low temperatures (starting from 100–120 °C) and leads to the formation of Si–C–Si and Si–C–C–Si units. This strengthens the polymeric network, since the Si–C and C–C bond are not affected by thermal depolymerization reactions such as transamination or exchange of Si–N bonds. Thus, higher ceramic yields as well as higher carbon contents are possible in the final ceramic materials [171]. Hydrosilylation can be also performed in presence of catalysts, which significantly increase the reaction rate [172].

Dehydrogenation of Si–H/N–H or Si–H/Si–H groups starts at temperatures of ca. 300 °C and leads to the formation of Si-N and Si-Si bonds as well as to hydrogen evolution.

The vinyl polymerization process occurs at higher temperatures and involves no mass loss. Upon UV light irradiation and in the presence of a photo initiator (such as 2,2-dimethoxy-1,2-diphenylethan-1-one), the vinyl polymerization process can occur at temperatures as low as ambient temperature, as it was also shown for other vinyl-substituted polymers [173].

Transamination reactions occur in a temperature range from *ca.* 200 °C to 400 °C and are associated with mass loss (*i.e.*, amines, ammonia, silazane (oligo)fragments), thus leading to a decrease in nitrogen content of the final ceramic materials. Since also redistribution reactions at silicon centers can occur and volatile silicon species, e.g., silanes, can evolve, the ceramic yield and the silicon content of the end ceramics consequently decrease.

### 3.2. Ceramization

The ceramization process of preceramic polymers involves the thermolysis and release of their organic groups at high temperatures (600–1000 °C) and consequently the organic-to-inorganic transformation of the preceramic materials into amorphous covalent ceramics [5,7,174]. Ceramics which are obtained by using this technique show, however, the disadvantage of high shrinkage and porosity. Greil *et al.* [175] investigated the effect of fillers dispersed within the pre-ceramic polymers on the shrinkage and porosity of the resulting ceramics. Using inert fillers, ceramic materials with less shrinkage can be obtained; the volume change upon polymer decomposition is accommodated however by the appearance of relatively large porosity [176,177]. The use of active fillers can compensate the shrinkage of the polymer matrix by appropriate expansion of the filler phase due to its reactions with the gaseous releases. Appropriate combinations of inert and active fillers might thus lead to dense materials with zero shrinkage, allowing for near-net-shape manufacturing [178,179]. An additional technique to produce dense PDC parts relates to an extensive cross-linking step which increases the ceramic yield of the polymer-to-ceramic transformation. This has been achieved for instance in the case of a polysiloxane, which was cross-linked by means of UV light irradiation. The residual porosity of the SiOC ceramic prepared upon pyrolysis of the UV light cross-linked green body was determined to be below 1% [173]. Also infiltration-pyrolysis cycles of pressureless monoliths have been shown to lead to materials with less residual porosity [180]. Beside pressureless techniques, dense PDC-based parts can be prepared by using pressure-assisted methods, such as uniaxial hot pressing (HP) [181,182], hot isostatic pressing (HIP) [183], or spark plasma sintering (SPS) [184,185], *etc*.

The conversion of preceramic polymers into ceramics involves complex processes which are difficult to investigate, due to the poorly defined structure of the preceramic materials as well as to the amorphous nature of the resulting ceramics. However, the use of solid state NMR, thermogravimetric analysis (TGA) coupled with differential thermal analysis (DTA) and *in situ* evolved gas analysis (EGA, *i.e.*, *in situ* FI-IR spectroscopy and mass spectrometry) as well as other modern structural characterization techniques, it is possible to rationalize the processes which occur during the ceramization of the preceramic polymers.

Pyrolysis of *polycarbosilanes* leads to amorphous silicon carbide-based materials at temperatures between 800 °C and 1000 °C upon release of gaseous species containing Si–H, Si–CH_3_, and Si–CH_2_–Si groups [165]. ^29^Si MAS‑NMR spectra of the materials annealed in this temperature range showed the presence of one single peak assigned to SiC_4_ units [186]. Additional competing decomposition processes lead to the formation of segregated carbon as well as dangling bonds within the amorphous ceramic. Consequently, the ceramics obtained from the pyrolysis of polycarbosilanes at 800 °C can be described as hydrogenated silicon carbide materials containing some segregated carbon [186,187]. As the annealing temperature exceeds 1000 °C, hydrogen is released and the crystallization of the amorphous material into cubic silicon carbide occurs.

The pyrolysis of polysiloxane-based preceramic polymers leads to the formation of silicon oxycarbide (SiOC) [51,188,189,190,191,192,193,194,195,196]. During the ceramization process, mainly the release of hydrocarbons and hydrogen takes place, in addition numerous distribution reactions between the Si–O, Si–C, and Si–H bonds [197]. They might lead to the evolution of low-molecular-weight silanes (at temperatures in the range from 400 and 600 °C) and consequently to a decrease of the ceramic yield. At higher temperatures (600 to 1000 °C), extensive cleavage processes of C–H, Si–C and Si–O bonds occur and furnish ceramic materials consisting of an amorphous silicon oxycarbide phase and residual segregated carbon [6,198,199,200,201,202,203]. Studies on the pyrolysis kinetics for the conversion polysilsesquioxane→silicon oxycarbide indicate that processes leading to evolution of hydrocarbons (methane) and hydrogen represent the main mechanism for the removal of carbon during pyrolysis [204]. This mechanism has been found to be of first-order. Furthermore, the reaction rate was found to directly correlate to the amount of the remaining carbon sites. Thus, the nanostructure/architecture of the silicon oxycarbide glassy network which results upon pyrolysis of the preceramic polysilsesquioxanes relies on the geometric configuration of the molecules in the cross-linked preceramic polymer.

Numerous polysiloxane compositions were studied as precursors for SiOC glasses [189,190,191,192,193,194,195,196]. It seems that the composition of the final SiOC glass can be controlled, since the O/Si molar ratio remains almost constant during the pyrolysis process. The tailoring of the polysiloxane composition leads to a minimization of the quantity of excess carbon in the final SiOC glass. This approach was applied by Soraru *et al.* [53] who introduced a proper amount of Si–H groups within the polysiloxane network to minimize the final free C content. Additionally, SiOC glasses with no excess of carbon (so-called “white” SiOC) were prepared via pyrolysis of a polysilsesquioxane in hydrogen atmosphere [205].

Numerous studies were performed in order to assess the conversion of polysilazanes into amorphous silicon carbonitrides (SiCN) [64]. The pyrolysis process of a polysilazane containing methyl and hydrido groups attached to Si, (–[H(Me)Si-NH]*_m_*–[Me_2_Si-NH]*_n_*–) [206,207], was investigated by means of MAS NMR and TGA/EGA. At temperatures exceeding 550 °C, ^13^C NMR and TG/MS investigations indicate reactions occurring between Si–H and Si–CH_3_ groups to form Si–CH_2_–Si units with methane evolution. Additionally, reactions involving N–H groups proceed to form SiN_4_–units by successive replacement of methyl groups and release of gaseous methane. With higher pyrolysis temperature, the number of Si–N or Si–C bonds successively increase, as observed by means of ^13^C and ^29^Si NMR spectroscopy [64]. For vinyl-containing polysilazanes, vinyl polymerization at moderate temperatures (250–350 °C) leads to the formation of carbon chains which subsequently might convert into sp^2^ carbon at higher temperatures [149]. Also, here the number of Si-N bonds in the ceramic materials increases with the temperature due to reactions of Si–H and Si–CH_3_ groups with N–H.

Corroborated TGA/EGA and FTIR spectroscopy studies on the ceramization transformation of a vinyl-substituted polyureasilazane revealed that at lower temperatures vinyl polymerization and hydrosilylation processes are responsible for the cross-linking of the precursor; whereas at higher temperatures transamination reactions occur, accompanied by ammonia gas evolution [208]. Further increase of the temperatures to 600–800 °C leads to a remarkable decrease of the amount of Si–H, Si–CH_3_ and N–H groups, accompanied by the evolution of hydrogen (dehydro-coupling reactions between Si–H and N–H bonds) and methane (reactions between Si–CH_3_ and N–H).

Studies on the structure of silicon carbonitride ceramics obtained via pyrolysis of polysilazanes showed that they consist of a single SiCN amorphous phase and some residual excess carbon [209]. Whereas the pyrolysis of polyorganosilylcarbodiimides leads to phase-separated SiCN ceramics, consisting of amorphous silicon nitride and segregated carbon [80,95,96,98,210]. Obviously, the different phase composition and nano/microstructure of polysilazane- and poly(silylcarbodiimide)-derived SiCN relies on the different thermal and pyrolytic behavior of the preceramic polymers. ^29^Si MAS-NMR and FTIR studies on the thermolysis of polymethylsilylcarbodiimide revealed at moderate temperatures (up to 600 °C) the occurrence of rearrangement and condensation reactions to form SiCNX_2_, SiCN_2_X, SiCN_3_, and SiNX_3_ sites (with X being NCN or NCHN). At higher temperatures, the decomposition of the carbodiimide units takes place [89,90,152]. In a first step the carbodiimide units rearrange into the isomeric cyanamide structure, followed by the release of C_2_N_2_ and N_2_ and the generation of amorphous Si_3_N_4_ [153].

## 4. Ceramic Nanocomposites with Tailor-Made Phase Compositions and (Micro)Structures

### 4.1. Tailor-Made Compositions from Single-Source Precursors

**Figure 7:**
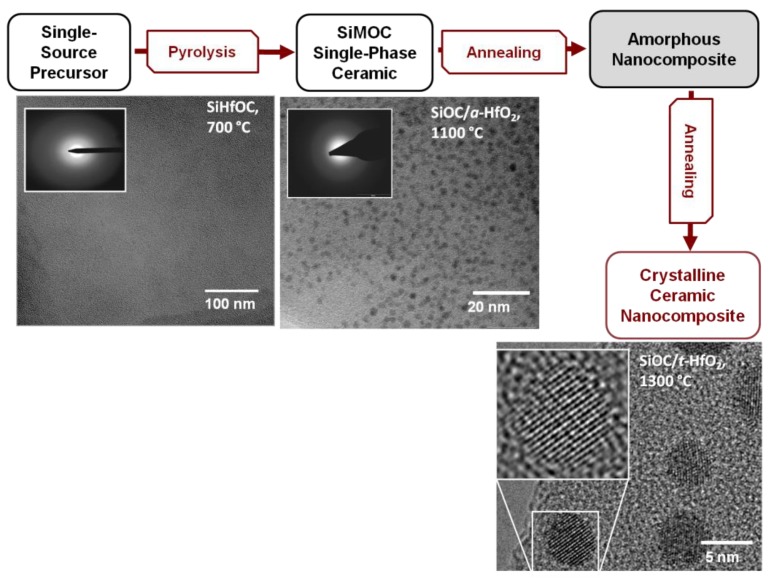
Single-source-precursor synthesis of polymer-derived ceramic nanocomposites (PDC-NCs).

The general synthesis strategy for PDC-NCs involves the preparation of suitable single-source precursors which can be converted in a first step into single-phase ceramic materials. Subsequent treatment of the single-phase materials (typically thermal treatment) leads to phase separation and crystallization processes which thus furnish nanocomposite materials with tuned phase compositions and microstructures. In Figure 7, the evolution of the microstructure of a SiHfOC-based material is shown. Pyrolysis at 700 °C delivers an amorphous, single-phasic SiHfOC material, which upon annealing at 1100 °C undergoes a phase separation process leading to an amorphous nanocomposite material consisting of amorphous hafnia nanoparticles homogeneously dispersed within a glassy SiOC matrix. Annealing at 1300 °C induces the crystallization of the hafnia precipitations and thus tetragonal hafnia nanoparticles finely dispersed within SiOC are obtained [4,9]. Within this subsection selected examples related to the ceramization of metal-modified silicon-containing preceramic polymers will be briefly introduced.

Pyrolysis of suitable alkoxysilanes, Si(OR)_4_, or polysiloxanes, –[Si(R)_2_–O]*_n_*, chemically modified with metal alkoxides was shown to give MO*_x_*/SiOC-based PDC-NCs, as reported for M = Al [179,211], Ti [54,58], Zr [203,212], Hf [201,213], *etc*. For M = Zr and Hf a single-phase SiMOC ceramic is obtained upon pyrolysis at rather low temperatures (*ca.* 700 °C), while at higher temperatures amorphous MO_2_ nanoparticles precipitate (800–1100 °C). Upon increasing the annealing temperature to 1300 °C, MO_2_ nanoparticles crystallize, forming microstructures comprised of tetragonal zirconia/hafnia particles finely dispersed within an amorphous SiOC matrix [201,203,213].

Metal-modified silicon oxycarbonitrides were also synthesized [154]; however, not only MO_x_/SiCNO nanocomposites (M = Ti [73], Zr [61], Hf [74,75]), but also M/SiCNO (for Cu [152], Ni [214]) and MSi_x_/SiCNO (Fe [215,216], Co [215], Pd [217]) were reported. In the case of MO*_x_*/SiCNO, a similar polymer-to-ceramic transformation as for MO*_x_*/SiOC was proposed (*i.e.*, formation of a single-phase amorphous SiMCNO at low temperatures and subsequent phase separation of MO*_x_*) [75].

Recently, the phase separation and crystallization of metal-modified silicon oxycarbides was systematically assessed [201,203,213,218,219]. Based on a case study related to the temperature-dependent evolution of the environment at the metal (*i.e.*, Hf) in a SiHfOC-based material prepared from a hafnium-alkoxide-modified polysiloxane [220], it was concluded that the Hf sites are coordinated only by oxygen, independently of which temperature was used for the preparation of the samples. This indicates that the amorphous single-phase SiMOC ceramics (M = metal) undergo phase separation and lead to the precipitation of an amorphous metal oxide (MO*_x_*) phase in a first step. Consequently, the (thermodynamic) stability of the MO*_x_* phase with respect to its carbothermal reaction (segregated carbon being typically present within the microstructure of the as-prepared amorphous SiMOC materials) was considered to be of crucial importance with respect to the evolution of the phase composition in SiMOC. Indeed the phase composition of SiMOC materials upon thermal treatment of metal-modified polysiloxanes can be rationalized via assessing the relative change in the free Gibbs energy of the systems M–MO*_x_* and C–CO (as segregated carbon is present within the microstructure of SiMOC, it is considered to determine the oxygen fugacity in the system) [221].

Based on thermodynamic data of the respective oxides, the phase composition of SiMOC ceramics upon annealing at high temperatures can be predicted. The prediction was shown to agree with the experimental results for SiMOC and also for SiMCNO ceramic composites. However, in addition to the stability of the oxides with respect to reduction, some other aspects were shown to be relevant while trying to predict the phase composition of SiMOC/SiMCNO nanocomposites, e.g., thermodynamic stabilization through the conversion of the metal oxide phase into silicates (for MO*_x_* being stable with respect to carbothermal conversion into M) or into silicides or carbides (for MO*_x_* not being stable against carbothermal reduction) [221].

A corroborated MAS NMR and electron microscopy study on the evolution of SiHfCNO indicate also in this case the fact that in a first step the hafnium-alkoxide-modified polysilazane used as a single-source precursor delivers upon pyrolysis an amorphous single-phase SiHfCNO ceramic, which in a subsequent step converts into an amorphous HfO*_x_*/SiCN nanocomposite. Annealing at higher temperatures leads to the crystallization of the hafnia phase into tetragonal HfO_2_ [75].

Also non-oxidic systems (e.g., SiMC, SiMCN, SiMBCN, with M being metal) were shown to be synthetically accessible upon using a similar approach [79,182]. Thus, SiHfC-based nanocomposites were prepared upon thermal conversion of a polycarbosilane modified with a tetrakis amido Hf complex; whereas SiHfCN and SiHfBCN nanocomposites were prepared from Hf-modified polysilazanes and polyborosilazanes, respectively. Interestingly, the high-temperature evolution of those systems seem to be also thermodynamically controlled. Thus, SiHfBCN-based single-phase materials convert into SiC/HfC/HfB_2_ ceramic nanocomposites via high-temperature exposition in argon atmosphere; whereas annealing in nitrogen leads to HfN(C)/Si_3_N_4_/SiBCN nanocomposites [79].

### 4.2. Hard-Template-Assisted Techniques towards Ordered (Micro)structures

As mentioned in Section 2, the synthesis of ceramic materials from preceramic polymers leads to several benefits as compared to other preparative methods; in particular based on the rheological and solubility capability of preceramic polymers. This offers for instance the opportunity to prepare porous materials. The introduction of pores (controlled or uncontrolled) in ceramic materials leads to hollow frameworks with modified physical and chemical properties in comparison to their dense counterpart. This extends the application potential of these materials in modern society and to particularly investigate their properties in the energy and environment fields.

**Figure 8:**
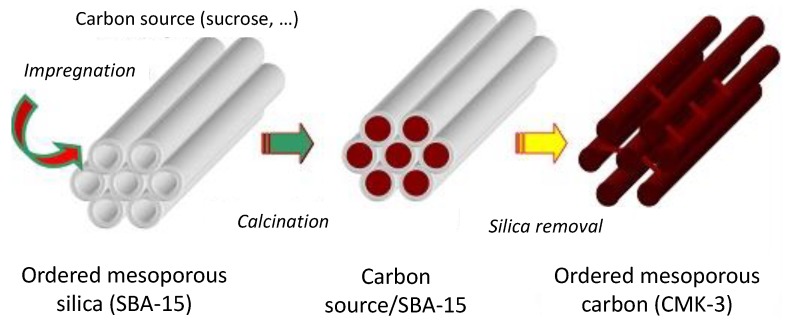
Nanocasting process: Toward the preparation of ordered mesoporous carbon.

We can classify porous materials into three categories based on the pore diameter (IUPAC classification): microporous (<2 nm), mesoporous (2–50 nm) and macroporous (>50 nm) [222]. The porosity can be disordered or ordered. It can be also hierarchical combining several types of porosity. Among these materials, the interest in the synthesis, characterization, modification/functionalization and application of ordered mesoporous materials has been developed dramatically over the last 20 years since the separate discovery of ordered mesoporous silica by Japanese scientists and Mobil researchers [223,224,225,226]. The important quantity of reports focused on ordered mesoporous materials is related to their particular characteristics which include their high specific surface area and pore volume within a relatively small volume of material, well-defined ordered mesostructure, structural capabilities at the scale of a few nanometers, tunable pore size, and varieties of the framework. This makes these materials attractive for applications in various fields, such as catalysis, adsorption and separation, drug storage and delivery, nanofabrication, sensors, photonics, energy storage and conversion, *etc.* [227]. The majority of mesoporous materials are of oxide type formed by a self-assembly process from combined solutions of sol–gel precursors (e.g., metal alkoxides) and structure-directing amphiphiles, usually block-copolymers or surfactants [222,228]. The most investigated oxide-based materials are siliceous materials. Despite the great success of this soft-templating method for oxides, it was rather challenging to apply this route to the synthesis of oxygen-free compounds satisfying the current technological needs in terms of thermo-structural and thermo-chemical stabilities. This is due mainly to the hydrolysis sensitivity and poor affinity with surfactants of the corresponding precursors. In addition, the generally higher temperatures which are applied to prepare non-oxide ceramics may cause the thermal breakdown of the structural integrity of the material. Only, few ordered mesostructures of non-oxide ceramics are reported using this strategy (see Section 4.3) [229,230,231]. A more convenient strategy has been proposed by Ryoo and co-workers in 1999 [232]. They reported the preparation of ordered mesoporous carbon using sucrose as the carbon source and the cubic (*Ia*3*d*) mesoporous silica molecular sieve referred to as MCM-48 as the template via three steps: (i) precursor infiltration inside mesochannels of the silica template; (ii) conversion of the precursor in the nanochannels of the silica template up to 800–1100 °C under vacuum or in an inert atmosphere; (iii) removal of the mesoporous silica template by dissolution typically in aqueous solution containing NaOH and ethanol. The resultant porous carbon material was referred to as CMK-1. This route is known as nanocasting because the entire manufacturing procedure is similar to the traditional casting method but on the nanoscale. This work demonstrated the feasibility of this nanocasting strategy on carbonaceous materials and this “hard template” concept illustrated in Figure 8 from hexagonal SBA-15 to prepare CMK-3 has been mainly applied for preparing mesoporous non-oxide ceramics. In this section, we have selected pioneering works of highly ordered mesoporous PDCs with tailored microstructures using different types of hard templates following the strategy depicted in Figure 8.

It should be mentioned that, like classical ceramics, PDCs can be roughly classified into amorphous and crystalline materials as well as composites and nanocomposites containing one or more phases distinguished from the matrix. As ordered mesoporous materials, the reports are mainly focused on silicon-containing amorphous PDCs.

**Figure 9:**
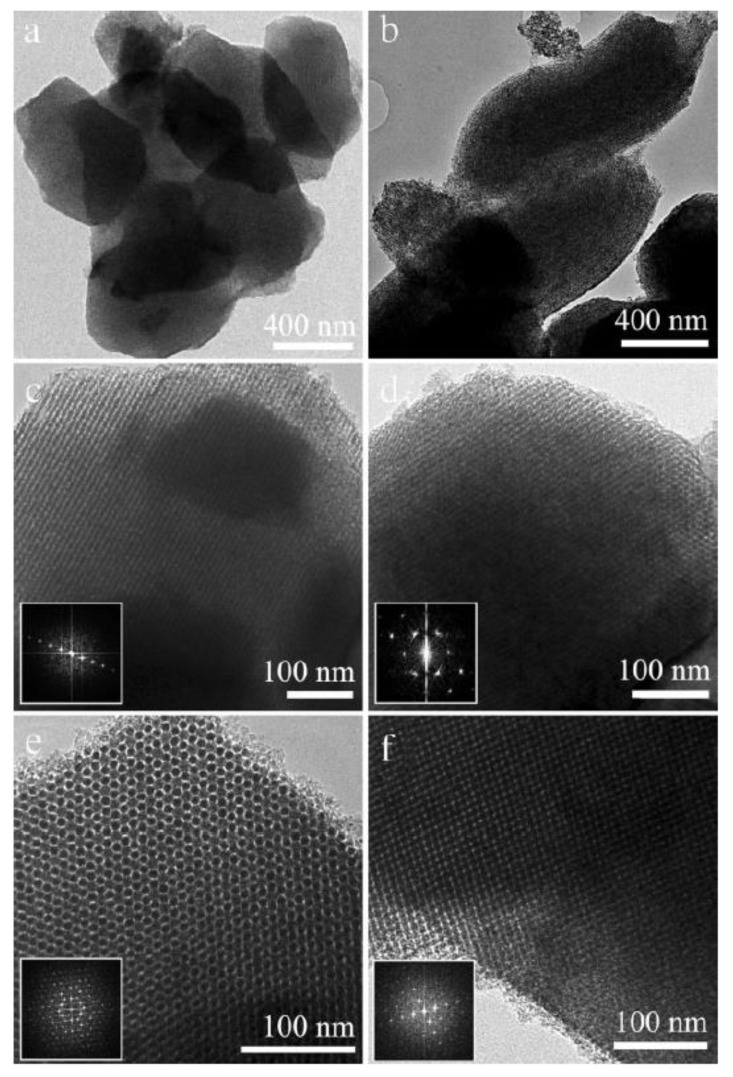
TEM images of ordered mesoporous SiC obtained by pyrolysis at 1200 °C from SBA-15 (silica-based Santa Barbara amorphous material No. 15) at low magnification (**a**,**b**) and high magnification (**c**,**d**) taken along the (**c**) (110) and (**d**) (100) directions. TEM images of ordered mesoporous SiC obtained by pyrolysis at 1400 °C from KIT-6 (Korea Advanced Institute of Science and technology No. 6) taken along the (**e**) (111) and (**f**) (531) directions. Insets show the corresponding Fourier diffractograms (reprinted with permission from reference [233]; Copyright Wiley).

The first example of ordered mesoporous PDCs concerns the preparation of highly ordered mesoporous silicon carbide (SiC) by nanocasting process using polycarbosilane (PCS) as a SiC precursor and mesoporous silica such as SBA-15 and KIT-6 as hard templates [233]. After successful impregnation, the PCS-to-SiC conversion was achieved at 1200–1400 °C and the resulting SiC–mesoporous silica composites were washed with 10 wt% aqueous hydrofluoric acid several times to remove the silica template. Depending on the nature of the templates, SiC nanowires in two-dimensional (2D) hexagonal arrays (*p*6*m*) as well as a three-dimensional (3D) bicontinuous cubic mesoporous SiC structures could be elaborated with high BET surface areas (460–720 m^2^·g^−1^) and uniform pore sizes (2–3.6 nm) (Figure 9). Heat-treatment of the sample to 1400 °C under nitrogen resulted in the decrease of the specific surface area (SSA) while crystallization of β-SiC occurred.

The possibility to modify the chemistry of precursors as well as their reactivity allowed tailoring the composition of ordered mesoporous materials. As an illustration, by selecting ammonia instead of inert atmosphere during the polymer-to-ceramic conversion, Zhao *et al.* [234] reported the preparation of ordered mesoporous amorphous silicon nitride from CMK-8 as a hard template using the reactivity of polycarbosilane (PCS) toward ammonia. Mesoporous silicon nitride samples displayed a 3D bicontinuous cubic mesostructure (*Ia*3h*d*) similar to KIT-6, a specific BET surface area of 384 m^2^·g^−1^, a large pore volume of 0.71 cm^3^·g^−1^, as well as a narrow pore size distribution at the mean value of 5.7 nm. A secondary impregnation-pyrolysis cycle could reduce the structural shrinkage and improve the mesostructural regularity. By changing the SiC precursors and selecting allylhydridopolycarbosilane, known as AHPCS, Kim *et al.* [235] prepared ordered mesoporous SiC from surface-modified SBA-15 by impregnation, pyrolysis to 1000 °C under nitrogen and silica etching using a 10 wt% HF solution in a 50:50 mixture of water and ethanol. Samples exhibited BET surface areas of 260 m^2^·g^−1^ with pore size of 3.4 nm. These examples show that the main strategy to develop the mesoporosity of PDCs and keep the high ordering of the pores is to generate PDCs for which the structure is predominantly amorphous; heat-treatments at higher temperatures involve both the transformation of the amorphous state into crystalline phases and tend to collapse the porous structure.

It is reported that the amorphous state of PDCs can be stabilized by increasing the number of constituents in the Si–C and Si–N systems. Monthioux and Delverdier [236] studied the crystallization behavior of various PDCs. Based on their TEM investigations, they reported that the nucleation of the excess free carbon phase, commonly present in PDCs is always the first crystallization phenomenon to occur, followed by the nucleation of SiC. Depending on the system studied, different onset temperatures for the occurrence of crystallization were monitored. While nucleation within the binary Si–C system started at temperatures as low as 900–950 °C, local crystallization within the ternary Si–C–N and Si–C–O systems was observed at 1100 °C and 1250 °C, respectively. The quaternary Si–C–N–O system remained amorphous up to 1400 °C. However, increasing the number of constituents within polymer-derived ceramics is not the only active parameter in PDCs to raise thermal stability against crystallization. Chemical composition, starting polymer and glass architecture as well as residual porosity have to be considered simultaneously.

**Figure 10:**
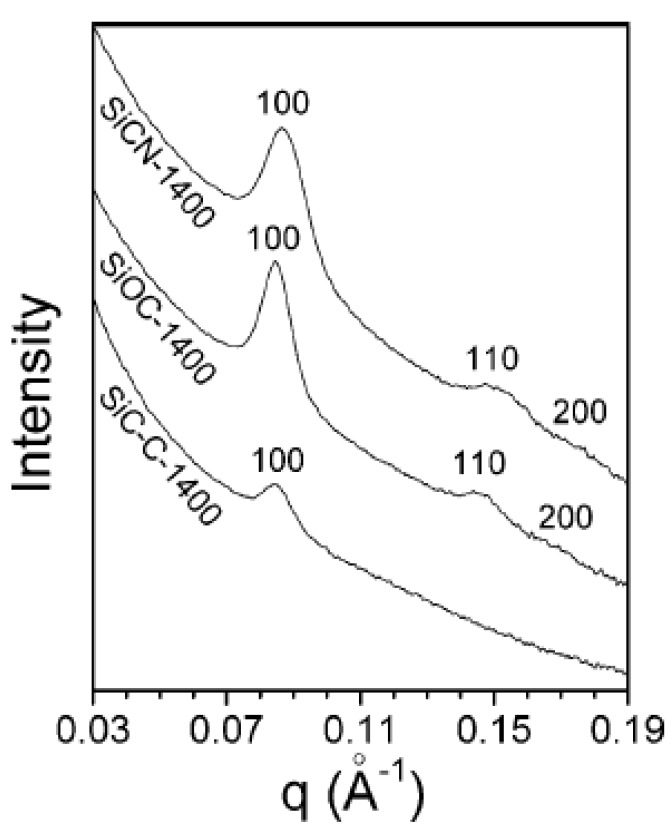
Small angle X-ray scattering (SAXS) patterns for ordered mesoporous SiC–C composites and derived SiOC-1400 and SiCN-1400 samples (reprinted with permission from reference [238]; Copyright 2007 American Chemical Society).

Based on these works, recent reports focused on multi-element (more than 2) materials with the objective to develop the thermal stability of ordered mesoporous PDCs. As an illustration, Kim *et al.* reported for the first time the preparation of highly ordered two-dimensional (2D) hexagonal and three-dimensional (3D) cubic mesoporous Si–C–N ceramics with high surface area (up to 472 m^2^·g^−1^), a narrow pore-size distribution and high thermal stability by nanocasting polycarbosilazane solutions into mesoporous carbon templates of the type CMK-3 and CMK-8 [237]. Interestingly, the BET surface areas of the mesoporous Si–C–N replicas were preserved up to 1000 °C in air. Within the same context, Zhao *et al.* developed another way which consisted to prepare through pyrolysis at 1400 °C firstly SiC within CMK-3 labeled SiC-C-1400, then in a second step, heat-treatment under air (500 °C) and ammonia (1000 °C) led to carbon elimination while ordered mesoporous Si–O–C (SiOC-1400) and Si–C–N (SiCN-1400) ceramics formed respectively as demonstrated by SAXS experiments (Figure 10) [238]. The ordered mesoporous Si–O–C and Si–C–N ceramics displayed high surface areas (200–400 m^2^·g^−1^), large pore volumes (0.4–0.8 cm^3^·g^−1^), and narrow pore size distributions (4.9–10.3 nm).

**Figure 11:**
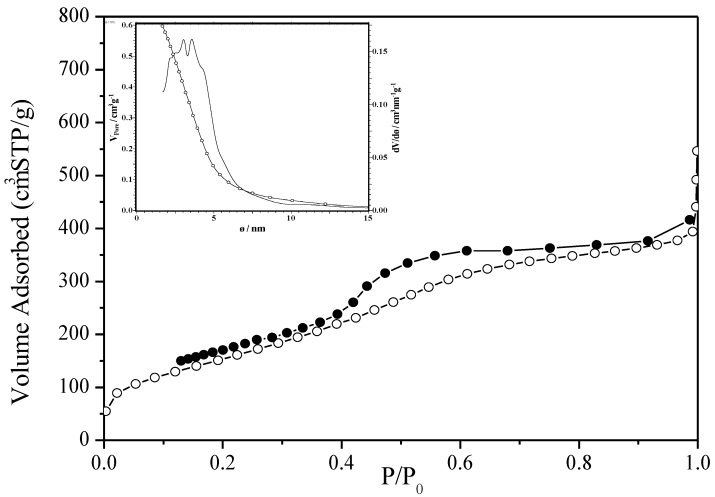
Nitrogen adsorption-desorption (○ and ●, respectively) isotherms of the ordered mesoporous SiBCN material. The pore size distribution curves are shown inset.

The introduction of boron in the Si–C–N system is known to shift the crystallization onset of the later to high temperature. Bill and co-workers [206,239] investigated the microstructure development of monolithic Si–(B)–C–N ceramics and they concluded that thin turbostratic B(C)N structures, finely dispersed within the amorphous matrix, acted as diffusion barriers preventing SiC and Si_3_N_4_ nucleation. In 2008, open, continuous, ordered 2D hexagonal mesoporous Si–B–C–N powders have been proposed by a double nanocasting approach using CMK-3 as template and boron-modified polysilazane of the type [B(C_2_H_4_SiCH_3_NCH_3_)_3_]*_n_* (C_2_H_4_ = CHCH_3_, CH_2_CH_2_) as preceramic polymer [240]. The polymer-to-ceramic conversion was achieved under ammonia up to 200 °C to cross-link the polymer via amine-exchange reactions then under nitrogen up to 1000 °C to generate a SiBCN-carbon composite. CMK-3 was subsequently removed through thermal treatment at 1000 °C in an ammonia atmosphere to generate ordered mesoporous SiBCN structures. Elemental analyses indicated the formation of ordered mesoporous powders with an empirical formula of Si_3.0_B_1.0_C_4.2_N_3.5_, whereas the nitrogen adsorption-desorption isotherm of the specimens showed a clear step at a relative pressure of about 0.5 attributed to capillary condensation in ordered mesoporous structures (Figure 11).

The corresponding specific surface area and the pore volume were calculated to be 600 m^2^·g^−1^ and 0.61 cm^3^·g^−1^, respectively. A narrow pore size distribution (around 3.4 nm) has been found. The material exhibited a relatively good thermal stability in air through heat-treatment to 1400 °C. Changing the Si–B–C–N precursor for a boron-modified polysilazane of the type [B(C_2_H_4_SiCH_3_NH)_3_]*_n_* (C_2_H_4_ = CHCH_3_, CH_2_CH_2_) with a higher ceramic yield allowed generating better ordered 2-D hexagonal frameworks [241]. Using a double impregnation cycle combined with a pyrolysis process up to 1000 °C in flowing nitrogen and a carbon removal step at 1000 °C for 3 h in ammonia and nitrogen atmospheres, the ordered mesoporous Si–B–C–N ceramic displayed high surface area (630 m^2^·g^−1^), high pore volume (0.91 cm^3^·g^−1^), and narrow pore size distribution (around 4.6 nm) with a thermal stability which extended up to 1180 °C under nitrogen. From the same polymer and using ordered mesoporous silica, periodic mesoporous silicoboron carbonitride (Si_3.0_B_1.0_C_3.9_N_1.8_) frameworks with *P6mm* hexagonal symmetry could be prepared after a double infiltration/thermal cross-linking cycle followed by a thermal process up to 1000 °C under N_2_ and a short chemical etching with dilute HF [242]. The ordered mesoporous Si_3.0_B_1.0_C_3.9_N_1.8_ ceramic displayed a specific surface area of 337 m^2^·g^−1^, a pore volume of 0.55 cm^3^·g^−1^ and a narrow pore-size distribution centered on 4.6 nm by N_2_ sorption with an amorphous network remaining stable during continuous heat-treatment to 1480 °C in a nitrogen atmosphere. The study developped in this paper showed that the use of carbonaceous template is preferred for preceramic polymers most probably due to the pore surface chemistry of carbonaceous template that involves more complete pore filling and the expected better chemical compataibility of carbon with ceramic precursors.

**Figure 12:**
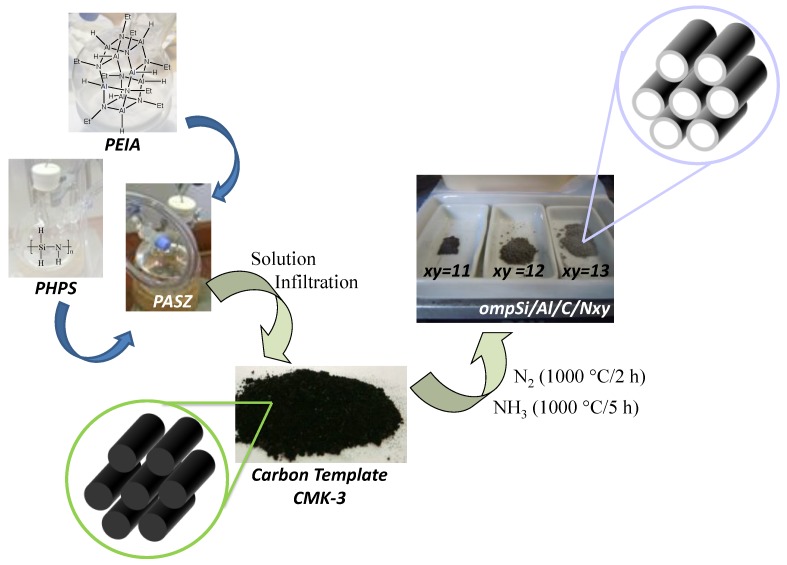
Procedure to prepare ordered mesoporous Si/Al/C/N ceramics by a polymer building block approach.

The Si–Al–C–N system is another interesting system. Indeed, the addition of Al to Si-based ceramics contributes to the improvement of their hydrothermal stability as illustrated through the addition of Al to Si/N/O systems forming Si/Al/O/N ceramics [243,244,245]. Similarly, An *et al.* reported that the addition of Al to Si/C/N(O) resulted into a non-parabolic oxidation curve (at *T* ≥ 1000 °C) which decreased more rapidly with time, down to a negligible level [246]. Authors suggested that the remarkably low oxidation rates of these materials were attributed to the lower permeability of the formed oxide layer to molecular oxygen which resulted from the incorporation of aluminum in the silica network. This passivating Si/O/Al layer is shown to hinder diffusion-controlled oxidation in the bulk. Within this context, the preparation of periodic mesoporous silicon-aluminum-carbon-nitrogen (Si/Al/C/N) frameworks with *P6mm* hexagonal symmetry using mesoporous carbon (CMK-3) as hard template and preceramic polymers containing both –[R_1_R_2_Si-N(R_3_)]*_n_*– and –[R_4_Al-N(R_5_)]*_n_*– backbones (with R_1_ = R_2_ = R_3_ = R_4_ = H and R_5_ = CH_2_CH_3_) as ceramic precursors was reported [247]. The preceramic polymers were prepared by blending poly(perhydridosilazane), PHPS, and poly(ethyliminoalane), PEIA, as precursors of silicon nitride/silicon (Si_3_N_4_/Si) and carbon-containing aluminum nitride (Al/C/N), respectively. The blended polymers with various and controlled Al:Si ratios were infiltrated into the porous structure of CMK-3 followed by a pyrolysis-template removal cycle performed under nitrogen at 1000 °C (2 h, ceramic conversion) then in an ammonia atmosphere at 1000 °C (5 h, template removal) (Figure 12).

This procedure resulted in the formation of periodic mesoporous Si/Al/C/N frameworks with surface areas of 182–326 m^2^·g^−1^, a pore size distribution of 4.1–5.9 nm and pore volumes in the range of 0.51–0.65 cm^3^·g^−1^. Amorphous materials did not exhibit weight change up to 1400–1470 °C in flowing nitrogen and their behavior in air up to 1000 °C (with dwelling time of 5 h) depended on the proportion of AlN and Si_3_N_4_ phases. Mesoporous materials are interesting because their pore size is similar to the dimensions of many molecules, which suggests that these materials could be potentially useful in separation, catalytic or nano-confinement processes (See Section 5.3). However, mesoporous PDCs are in general produced as powders which have some difficulties in practical use and as a consequence a limited application. Practical applications require that the mesoporous material is available in macroscopic form such as monolith.

**Figure 13:**
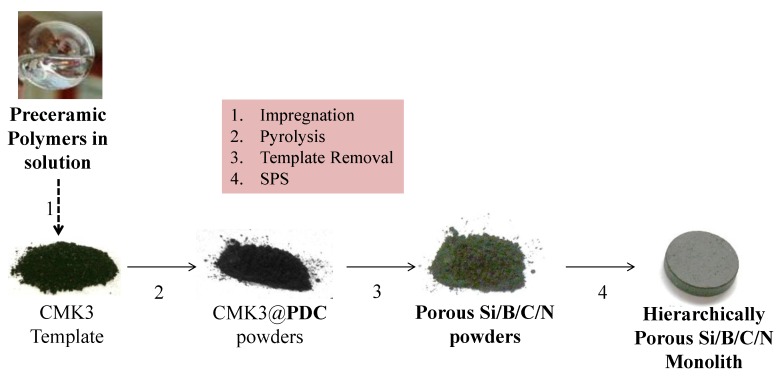
Overall synthetic path employed to generate hierarchically porous Si/B/C/N monoliths coupling the PDCs route with nanocasting and spark plasma sintering (SPS) processes.

The current technology for producing porous non-oxide ceramic monoliths involves extrusion or pressing powders together with sacrificial and sintering additives into an engineering shape, removal of all sacrificial additives and finally sintering at high temperature. Sintering additives are usually added to impart high mechanical strength. As an alternative, the elaboration of monolith-type mesoporous PDCs can be achieved by impregnation of silica or carbon foams [248]. As a better alternative, the elaboration of ordered mesoporous powders through the PDCs route may be combined with an approach that adopts the convenience and flexibility of powder-based processes such as spark plasma sintering (SPS). This has been performed on ordered mesoporous Si/B/C/N powders displaying *P6mm* hexagonal symmetry and their processing led to hierarchically porous Si/B/C/N monoliths through SPS without the use of any sintering additives (Figure 13) [249,250].

The coupled approach allowed obtaining robust monoliths with surface areas of 123–171 m^2^·g^−1^, mesopore diameters of ca. 6.2–6.5 nm and total pore volumes varying from 0.25 to 0.35 cm^3^·g^−1^. The characteristics of the monoliths are related to the use of ordered mesoporous powders as starting materials and to the control and the tailoring of the pore size and the connectivity over a relatively wide range of length scales through the parameters of sintering. SiBCN monoliths displayed porosities from 59% to 69%, a meso-/macroporosity which differed from starting powders. The use of SPS inhibited grain growth during sintering leading to materials which retained the intrinsic properties of the pristine powders.

### 4.3. Self-Assembly Strategies Based on Preceramic Block Copolymers and Particles

The self-assembly of polymers such as block copolymers or polymeric spherical particles is a feasible tool for the formation of nanostructured (hybrid) materials for a manifold of different potential applications. Compared to the previous section predominantly dealing with hard-templating methodologies, polymer-based templating strategies for the preparation of advanced ceramic are addressed within this section. Two self-assembly techniques of preceramic polymers are of special interest: (i) the self-assembly of block copolymers featuring a ceramic precursor as integral part of the polymer chain for at least one block segment and (ii) colloidal crystallization of polymeric spherical (hybrid) nano-particles. In the recent past, both concepts have attracted significant attention in order to generate ordered ceramic (composite) nano-structures.

Block copolymers which consist of two or more polymer segments covalently connected to each other are capable of undergoing microphase separation. Already in the case of two different block segments, various structures, e.g., spheres, cylinders, lamellae and co-continuous structures as well as porous structures at the nanoscale can be obtained only by variation of the volume fraction of underlying block segments [251,252,253]. On this account, the exploitation of block copolymer self-assembly in order to template inorganic materials has attracted significant attention. Besides polymeric parameters such as the overall molar mass or the polymer constitution, different other factors, e.g., temperature, solvent vapor or pressure strongly affect the microphase separation. Moreover, block copolymer assembly can be directed by the application of flow fields and electric or magnetic fields [254]. Recent strategies focus on guiding the self-assembly of such polymers on patterned substrates and in confinements [255,256]. Within the block copolymer self-assembly strategies, different approaches for the preparation of ceramic materials inclusively nanocomposites are known. Excellent reviews within that field are given, e.g., by Orilall and Wiesner [257] and other authors [258,259,260,261,262].

Although this review focuses on preceramic polymers, the self-assembly of block copolymers prior to the selective removal of one microphase-separated block segment will be exemplarily discussed focusing on the preparation of block copolymer templated ceramics.

Separated block domains of these nano-structures can be selectively removed. In a subsequent step, the residual nano-structure can be backfilled with inorganic precursors followed by etching or calcination in order to remove the second block copolymer segment. For example, Hsueh *et al.* [263] reported the preparation of bicontinous anatase (TiO_2_) ceramics using this double-templating approach. The authors took advantage of the removal of the microphase-separated poly(l-lactide) segment by hydrolysis followed by backfilling of a titanium alkoxide precursor. Calcination yielded ordered and porous titanium oxide with photocatalytic properties. This route can also by applied for the preparation of porous silica materials featuring a very low refractive index [264]. A bicontinous microemulsion of polyethylene (PE), poly(ethylene-*alt*-propylene) (PEP), and poly(ethylene-*block*-ethylene-*alt*-propylene) (PE-PEP) has been advantageously used for the preparation of porous structures after selective removal of PEP [265]. Backfilling of the voids with poly(ureasilazane) (Ceraset) and thermal treatment yielded 3D continuous SiCN ceramics. The preparation of different mesoporous rare-earth oxide ceramics using evaporation-induced self-assembly of block copolymers has recently been reported [266].

As described above, the multi-step strategy based on the self-assembly of organic block copolymers followed by removal of one block segment is a powerful method. As another feasible route, the block copolymer *co-assembly* and hence a direct incorporation of preceramic polymers or inorganic particles will now be described.

**Figure 14:**
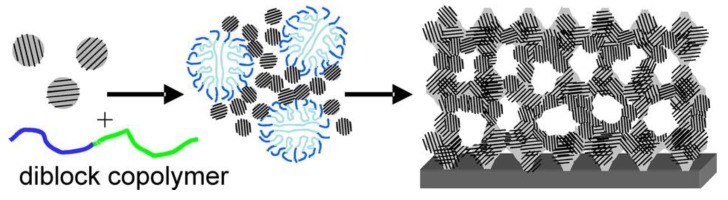
Schematic illustration for the preparation of porous metal oxides by using the evaporation induced self-assembly (EISA) of a dispersion consisting of diblock copolymers and inorganic particles in the first step followed by thermal treatment. By this general methodology mesoporous ceramics can be obtained (reprinted with permission from reference [267]; Copyright 2012 American Chemical Society).

Recently, Rauda and coworkers reported a general method for the production of templated mesoporous materials based on preformed nano-crystal building blocks [267]. Here, soluble diblock copolymers mixed with inorganic particles were capable of undergoing an evaporation-induced self-assembly (also referred to as EISA process) (Figure 14). After thermal treatment, nano-porous metal oxides, e.g., manganese oxides could be obtained [267]. Poly(dimethylsiloxane)-*block*-poly(ethylene oxide) (PDMS-*b*-PEO) has been used as template for the incorporation of methylphenylsiloxane (MPS) in order to fabricate porous MPS/PDMS composites featuring a high framework stability [268].

Block copolymer *co-assembly* of organic block copolymers and a preceramic polymer such as poly(ureamethylvinylsilazane) (PUS)—also referred to as Ceraset—is reported by Wiesner and coworkers [229,230,269]. There, the diblock copolymers poly(isoprene-*block*-dimethylaminoethyl methacrylate) or poly(isoprene-*block*-ethylene oxide) can be used as structure-directing agent for the commercially available silazane-based PUS for the preparation of high temperature SiCN ceramic materials [229,230,269]. The affinity of PEO to PUS has also been investigated by Wan *et al.* [270] Upon pyrolysis of the microphase-separated PUS/block copolymer blend (polybutadiene-*block*-PEO), SiCN-based non-oxide ceramics could be obtained.

**Figure 15:**
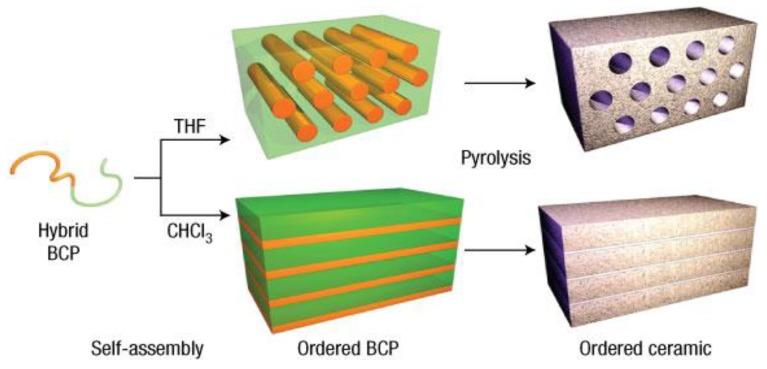
Conversion of a self-assembled hybrid block copolymer featuring an inorganic precursor segment into ordered ceramic structure by pyrolysis (reprinted with permission from Nature Publishing Group) [230].

Another efficient strategy for the controlled buildup of ceramic nanostructures focuses on the self-assembly and pyrolysis of preceramic/organic block copolymers wherein the inorganic block segment is directly converted into the ceramic material after thermal treatment. Compared to previously described methodologies, inorganic monomers or metal-containing organic monomers were polymerized in a controlled manner to gain access to well-defined block copolymers. The basic concept is schematically depicted in Figure 15 based on the work of Malenfant *et al.* [230]. The authors took advantage of the self-assembly and pyrolysis of poly(norbornene)-*block*-poly(norbornene decaborane) in which the borane-containing block segment acted as ceramic precursor. Thermal annealing of the block copolymer and pyrolysis at 400–1000 °C in the presence of ammonia was evidenced to maintain the pristine block copolymer morphology for the final boron nitride ceramic. Interestingly, cylindrical morphologies of the preceramic block copolymer could simply be transferred into a lamellar morphology by changing the solvent in the annealing step [230].

Within the field of block copolymers featuring a preceramic polymer segment, especially silicon-based precursors have spurred much interest. Nghiem *et al.* [271] reported the synthesis of polycarbosilane-*block*-polystyrene by using living anionic polymerization protocols. Well-ordered SiC-based ceramic materials with a high content of micro-pores could be obtained. Polycarbosilane-based block copolymer micelles have been utilized after platinum-catalyzed cross-linking reaction for the preparation of silicon-containing ceramics [272]. Nguyen *et al.* [273] reported a feasible preceramic diblock copolymer consisting of acrylated poly(vinylsilazane)-*block*-poly(methyl methacrylate) (PVSZ-*b*-PMMA) as precursor for a SiCN-based ceramic patterning. Pyrolysis at 1200 °C yielded mesoporous SiCN ceramics with pore-sizes between 6 and 9 nm dependent on the applied cross-linking strategy.

Fascinating co-continous double gyroid and inverse double gyroid structures of block copolymer architectures consisting of polyisoprene and poly(pentamethylsilylstyrene) have been synthesized and used for the preparation of nano-porous or nano-relief structures as reported by Chan *et al.* [274]. Very recently, self-assembled block copolymers based on silsesquioxane-containing segments have been used for the preparation of ceramic-metal composites as reported by Li and coworkers [275]. For this purpose, platinum nanoparticles were added prior to the solvent-casting step for the phase separating block copolymer. UV-assisted ozonolysis both revealed maintenance of the hybrid block copolymer morphology as well as the formation of ordered inorganic nanocomposites [275]. Silicon oxy carbide nano-ring arrays have been produced after self-assembly and oxygen plasma treatment of polystyrene-*block*-poly(dimethylsiloxane) thin films [276]. The authors expect these interesting structures to be excellent mask materials for patterning applications.

Noteworthy, the usability of block copolymers is not limited for the formation of ceramic films or particles but also for the preparation of SiCN fibers. As an example, Pillai *et al.* [277] reported the reaction of high density polyethylene bearing a reactive hydroxyl moiety with a commercially available polysilazane (HTT1800). Self-assembly from different solvents either led to a cylindrical or lamellar morphology which could be converted into the corresponding SiCN ceramic fibers [277].

As already mentioned in Section 2.2, a manifold of iron-containing ceramics can be obtained by using metallopolymers. These metal-containing block copolymers can be used for the preparation of ceramic structures at the nano-scale as reported, e.g., by the group of Manners. Within that field, especially polyferrocenyldimethylsilane-based (PFS) block copolymers with the intrinsic capability of crystallization have shown a large potential as preceramic polymers replicating the micro-morphologies in the final ceramic material. Rider *et al.* [129] reported the formation of ordered nano-domains by self-assembling polystyrene-*block*-poly(ferrocenylethylmethylsilane) (PS-*b*-PFEMS) in the bulk state or in thin films. Magnetic arrays consisting of C/SiC-containing iron nano-particles were obtained after pyrolysis of the ferrocene-containing block copolymers. Ceramic surface-relief gratings (SRG) based on the ceramization of PFS block copolymers have been reported [278]. Ceramic SRGs are expected to be useful for templating and data storage materials. In earlier studies magnetic nano-lines, nanoscopic iron oxide patterns, separation tunable arrays and cobalt magnetic dot arrays were accessible by using PFS-based block copolymers as preceramic polymers [279,280,281,282].

Besides PFS-based block copolymers, also some other ferrocene-containing polymers based on methacrylate derivatives and vinylferrocene were capable of forming fascinating micro morphologies [283,284,285]. As an example of iron oxide as the final ceramic material, Tang and coworkers investigated the self-assembly and template synthesis of triblock copolymers with a poly(2-(methacryloyloxy)ethyl ferrocenecarboxylate) (PFcMA) block for the preparation of ordered iron oxides [135].

The self-assembly of preceramic polymer and core/shell architectures is another powerful method in order to generate well-defined 3-dimensional (hierarchical) ceramic materials after pyrolysis. It has to be borne in mind that 3D ceramic architectures are typically derived by previously mentioned technique, e.g., sol–gel and infiltration routes. Within this section, some examples for preceramic polymers or polymeric single-source precursors as integral part of colloidal architectures or spherical template route will be addressed. In this particular case, the self-assembly focusses on monodisperse particles featuring the intrinsic capability of colloidal crystallization. Based on such almost monodisperse polymeric colloidal micro- and nanoparticles, fascinating functional materials especially for optical applications can be obtained [286,287,288,289,290]. In general, colloidal crystals can be prepared from their particle dispersions by various techniques of deposition, spin coating or by using the melt-shear technique [291,292,293].

As mentioned above, PFSs were proven suitable preceramic polymers and also 3-dimensional iron-containing ceramics have been prepared from these precursors. Ozin and Manners *et al.* [294] have shown a gravity sedimentation route of silica spheres followed by thermal polymerization of a [1]silaferrocenophane monomer. Thermal treatment of the preceramic ferrocene polymer led to conversion into predominantly magnetic γ-Fe_2_O_3_. Well-defined magnetic ceramic opals with high structural periodicities were accessible by this method [294]. SiC inverse opals were accessible by a soft templating route using monodisperse polymeric spheres followed by infiltration with a silylene-acetylene preceramic polymer [295]. These remarkably stable SiC photonic crystals are expected to be feasible for optical applications in harsh environments.

## 5. Properties of Ceramic Nanocomposites

### 5.1. Structural Properties

#### 5.1.1. High-Temperature Stability towards Crystallization and Decomposition

*Silicon oxycarbides* are amorphous ternary ceramics which can be described as consisting of a glassy network of SiO*_x_*C_4−*x*_ tetrahedral [296,297] which formally can be considered as the result of the incorporation of carbon into silica glass, which has been shown in numerous studies to strongly affect its crystallization resistance at high temperatures [5]. Thus, silicon oxycarbides remain predominantly amorphous up to temperatures of 1300–1350 °C; whereas silica is prone to crystallization at temperatures as low as 1200 °C. The remarkable crystallization resistance of SiOC ceramics has been considered to rely on the incorporation of carbon into the amorphous network. Additionally, excess carbon (which, typically is present within the SiOC microstructure) has been also shown to affect the crystallization behavior of SiOC [297].

At temperatures beyond 1100 °C amorphous silicon oxycarbides undergo a phase separation process to form amorphous nanocomposites consisting of silica, silicon carbide and segregated carbon [53,298,299]. The phase separation in SiOC materials was extensively investigated by means of ^29^Si MAS NMR as well as high resolution TEM (HR-TEM) and relies on rearrangement processes occurring within SiO*_x_*C_4−*x*_ tetrahedra. ^29^Si MAS NMR spectra of SiOC materials as prepared at temperatures of *ca.* 1000–1100 °C exhibit a mixture of SiO_4_, SiO_3_C, SiO_2_C_2_, SiOC_3_ and SiC_4_ sites, which reflects the situation present within the glassy SiOC network. However, upon annealing at higher temperatures, significant changes of the network occur. Thus, nearly exclusive SiO_4_ and SiC_4_ environments are observed, indicating the phase separation of the SiOC to deliver amorphous silica-rich phase beside amorphous silicon carbide nano sized precipitations and segregated carbon [53,300].

Subsequent to the phase separation process, the crystallization of the phase-separated silicon carbide takes place at higher temperatures in the SiOC materials. In silicon oxycarbides annealed at temperatures of *ca.* 1200–1300 °C, nano sized precipitations of β-SiC are dispersed within a silica-rich matrix. Interestingly, the amorphous silica-rich matrix remains amorphous up to high temperatures, (e.g., no cristobalite formation was observed); this relies probably on the low interfacial energy between β-SiC and the amorphous silica-rich phase [301]. In a recent study, the presence of cristobalite was observed upon long-term annealing SiOC-based ceramics at temperatures between 1300 and 1400 °C. Its formation was explained as a result of the presence (or generation) of inner surfaces within the material, which probably promotes the devitrification of silica [302]. Moreover, a case study indicates that probably the molecular architecture of the precursor might also affect the formation of cristobalite: linear polysiloxanes convert into SiOC materials with very high crystallization resistance; whereas cristobalite was detected in silicon oxycarbides derived from cyclic precursors [192].

Annealing of SiOC at temperatures exceeding 1350–1400 °C, leads to its gradual decomposition due to two decomposition processes: at temperatures of 1350–1400 °C the carbothermal reaction of the phase‑separated silica with excess carbon occurs, and is accompanied by the formation of β-SiC and gaseous CO. At higher temperatures (*i.e.*, above 1500 °C), silica can react with SiC to gaseous silicon monoxide and CO, thus leading to a severe decomposition of SiOC [300,301].

The incorporation of additional elements into the network of silicon oxycarbides has a substantial effect on their decomposition and crystallization behavior. SiBOC ceramics were shown for instance to be less stable towards decomposition as compared to their boron-free counterparts and thus silicon carbide was found to crystallize SiBOC at lower temperatures [303,304,305,306].

The incorporation of transition metals such as Zr or Hf into SiOC was shown in several studies to drastically improve its high-temperature behavior with respect to decomposition and crystallization [6,201,203,307]. This relates to the fact that at temperatures exceeding 1400 °C (*i.e.*, in conditions which lead to the carbothermal degradation of SiOC) the MO_2_ phase present within the microstructure of the metal-modified SiOC undergoes a solid state reaction with the phase-separated silica to generate crystalline MSiO_4_. This was shown not only for SiZrOC and SiHfOC [201,203], but also for SiAlOC (crystallization of mullite at *T* > 1300 °C) [211], SiMnOC (crystallization of MnSiO_3_ already occurs at *T* ≈ 1100 °C) or SiLuOC (Lu_2_Si_2_O_7_ crystallization at *T* > 1300 °C) [221]. The preferred reaction of silica with the metal oxide phase is responsible for the improved high-temperature stability of SiMOC (M = Zr, Hf) with respect to decomposition. Due to the fact that silica reacts with MO_2_ to form MSiO_4_, its carbothermal reaction with excess carbon which leads to the crystallization of β-SiC and to CO release (*i.e.*, mass loss) is suppressed [201,203].

Also in *silicon carbonitride*-based ceramics decomposition and crystallization correlate to the chemical composition, architecture and chemical homogeneity of the amorphous SiCN network [5]. The stability of polysilazane-derived SiCN ceramics is determined by the reaction of silicon nitride with excess carbon which leads to silicon carbide and nitrogen gas release at temperatures exceeding 1484 °C [308]. SiCN ceramics which do not have excess carbon in their microstructure show a thermal stability which is greatly improved as it is now limited by the thermal decomposition of silicon nitride (occurring in 1 bar N_2_ atmosphere at *T* > 1841 °C [309]).

As already mentioned in Section 4.2, the incorporation of boron into the Si–C–N ceramic network was shown to significantly increase its thermal stability and crystallization resistance [310]. SiBCN ceramics remain amorphous up to *ca.* 1700 °C and show no significant decomposition up to 2000 °C [311]. Their extraordinary thermal stability is believed to rely rather on kinetic than thermodynamic reasons. Structural disorder in Si–B–C–N ceramics, which results in increased activation energies of both crystallization and carbothermal degradation processes, is thought to be responsible for the thermal stability of these materials. Furthermore, the presence of turbostratic B*_x_*C*_y_*N*_z_* phases within the microstructure of SiBCN are considered to kinetically stabilize the crystalline, phase-separated Si_3_N_4_ (which was still detected in SiBCN materials annealed at temperatures as high as at 2200 °C) and thus provide stable SiBCN composition up to very high temperatures [312,313]. The kinetics of the crystallization of Si_3_N_4_ in SiBCN ceramics [314] as well as the effect of the boron content on the crystallization behavior of SiBCN [315] was reported. Thermodynamic data of amorphous SiBCN ceramics determined by high-temperature oxide melt solution calorimetry were recently also reported and indicate that SiBCN might be considered as being thermodynamically stable with respect to the crystalline components SiC, Si_3_N_4_, BN and graphite [316]. As the thermodynamic stability of SiBCN decreases upon increasing the boron content, the effect of boron on the Si_3_N_4_ crystallization is thought to be exclusively a kinetic effect.

The incorporation of metal such as Al [246,317], Y [72,76], Ti [73], Zr [61,62], or Hf [6,74,318] into SiCN was reported in several studies. Amorphous SiMCNO ceramics are obtained via pyrolysis of metal-alkoxide-modified polysilazanes and undergo phase separation processes at high temperatures to generate amorphous metal oxide nanoparticles dispersed within glassy SiCNO matrix. Annealing at higher temperatures leads to the crystallization of the metal oxide nanoparticles.

#### 5.1.2. High Temperature Oxidation and Corrosion Behavior

Polymer-derived ceramic nanocomposites have been investigated in the last two decades concerning their high temperature oxidation behavior. Typically temperatures exceeding 1000 °C and oxidizing environments such as air or combustion atmosphere are applied. Within this context ternary and multinary materials based on the Si–M–O–C and Si–M–C–N–O systems (M = B, Al, Zr) were tested and were shown to exhibit passive oxidation behavior [319], thus they can be considered as behaving to some extent like silica formers, e.g., silicon, metal silicides, SiC or Si_3_N_4_.

Polymer-derived SiC ceramics exhibit regular (passive) oxidation behavior in pure oxygen or dry air atmosphere, *i.e.*, a parabolic growth of a pure silica layer in the temperature range from 800 to 1400 °C. The activation energy of the process was found to be *ca.* 100 kJ/mol, indicating the inward diffusion of oxygen through the growing silica layer (accompanied by the outward CO diffusion) as the rate-limiting mechanisms. This behavior is similar to that observed for silicon or pure silicon carbide. The amorphous silica layer crystallizes into cristobalite at temperatures above 1200 °C; however, the formation of cristobalite was shown to not significantly affect the oxidation rates [320,321], despite the diffusion of oxygen through cristobalite is lower than that through glassy silica [322].

SiOC ceramics exhibit parabolic oxidation behavior [323], with oxidation rates strongly depending on the content of the segregated carbon present within their microstructure. Thus, SiOC ceramics with high free carbon content oxidize consequently faster. However, the activity of the free carbon in SiOC is considered to be less than unity; therefore, passive oxidation in the Si–O–C system is possible even for the case of carbon-rich compositions.

Studies on the oxidation behavior of dense SiCNO ceramic materials revealed the formation of a dense and continuous oxide layer with a sharp oxide/SiCN interface and parabolic kinetics from 800 °C to 1400 °C [320]. The parabolic constants and the activation energies were found to be similar with those obtained for silicon carbide and silicon nitride [319].

In the case of SiBCN-based ceramics, exceptionally low oxidation rates were reported [324,325]. However, there are several aspects which were not systematically considered and which probably led to a strong underestimation of the oxidation rates: formation of low-viscosity borosilicate passive layer, volatilization of boron (sub)oxides, *etc*. Moreover, the role of the B incorporation within SiCN on its high-temperature oxidation behavior is not well understood so far. The addition of aluminum (SiAlBCN) [326] or hafnium (SiHfBCN) [327] was shown to negatively affect the oxidation behavior of SiBCN, *i.e.*, higher oxidation rates were reported.

The modification of Si–C–N–(O)-based PDCs with aluminum results in a non-parabolic oxidation behavior at *T* of *ca.* 1000 °C. At *T* = 1400 °C, a stable parabolic behavior was observed for *t* > 20 h, with oxidation rates about one order of magnitude lower than those reported for Al-free Si–C–N [246,328,329] This behavior was considered to be a consequence of the formation of an Al-containing silica scale, which induces a strong decrease of the oxygen diffusivity if compared to that of the Al-free silica [330].

The Zr addition into Si–C–N–O was shown to reduce the parabolic oxidation rate as compared to those of Si–C–N–O and was related to both the lower content and the lower activity of the segregated carbon in the Zr-containing system [62].

Recently, oxycarbide- and carbonitride-based PDC-NCs were studied with respect to their hydrothermal corrosion behavior [78,181].

Silicon oxycarbide-based ceramics exhibit an active corrosion behavior over the whole investigated temperature range (up to 250 °C), *i.e.*, silica being leached out of the samples. However, the corrosion rates of the SiOC ceramic materials were found to be remarkably lower than those of silicon carbide and were comparable to values reported for silicon nitride. Thus, a corrosion rate of 0.13 mg·cm^−2^·h^−1^ was determined for the SiOC sample upon corrosion at 250 °C, being *ca.* 5 orders of magnitude smaller than the rates obtained for SiC ceramics corroded under similar conditions. This fact might be related to the presence of a relatively high amount of segregated, “free” carbon within the microstructure of the SiOC sample (*ca.* 37 mol%), which is not affected by hydrothermal corrosion. Interestingly, Zr- and Hf-incorporation within SiOC was shown to lead to a significant improvement of its corrosion resistance. This was attributed to the presence of the ZrO_2_/HfO_2_ phase within the PDC-NCs, which exhibits an extremely low solubility in water under the investigated conditions. Thus, the finely disperse oxide nanoparticles acts as a “reinforcing” phase with respect to hydrothermal corrosion and are most probably the reason for the significant improvement in the corrosion resistance of SiZrOC/SiHfOC as compared to that of SiOC. Interestingly, the SiOC matrix was found to effectively suppress the corrosion-induced phase transformation of the tetragonal ZrO_2_/HfO_2_ phase into monoclinic ZrO_2_/HfO_2_, which is a well-known problem in the case of zirconia and hafnia materials exposed to hydrothermal conditions. Thus, the outstanding hydrothermal corrosion behavior of the investigated Zr-/Hf-containing SiOC ceramic nanocomposites relies on a unique synergistic effect between the reinforcing role of the ZrO_2_/HfO_2_ phase and the protection of the tetragonal oxide precipitates from corrosion-induced phase transformation through the SiOC matrix [181].

Ceramic matrix composites consisting of carbon fibers embedded within a SiCN and SiHfBCN matrix were also investigated concerning their behavior in hydrothermal conditions [78]. The preparation of the CMCs was performed via polymer infiltration and pyrolysis process (PIP) of 2D carbon fabrics with a polysilazane and an Hf- and B-modified polysilazane, respectively. Interestingly, it was observed that the C_f_/SiCN CMCs exhibits a weak fiber-matrix interface; whereas the interface between the carbon fibers and the SiHfBCN matrix was strong. Consequently, the mechanical behavior of the C_f_/SiCN samples was shown to be affected by the incorporation of Hf and B. The hydrothermal corrosion of the prepared CMCs revealed that C_f_/SiHfBCN samples exhibit better resistance as compared to C_f_/SiCN due to the improved kinetics upon Hf and B incorporation. Additionally, a tight C_f_/matrix interface (which is rather disadvantageous for appropriate mechanical behavior) was found to be beneficial for an improved corrosion behavior in C_f_/SiHfBCN [78].

#### 5.1.3. High Temperature Creep Behavior

Polymer-derived ceramics and ceramic nanocomposites were extensively investigated in the last 15 years with respect to their (thermo)mechanical properties. Especially, their high-temperature creep behavior was shown to be outstanding, thus this class of ceramic nanocomposites show near-zero steady state creep even at temperatures exceeding 1000 °C [5].

The creep behavior of silicon oxycarbides was shown to rely on viscous flow, as it is also the case for silica glasses [331,332,333,334,335]. As silicon oxycarbides can be formally considered as silica-based glass having carbon incorporated within the glass network, it was expected that their mechanical properties and refractoriness will be improved if compared to silica [332,333,335]. This effect was expected as a consequence of formally replacing bivalent oxygen atoms within the glassy network with tetravalent carbon atoms, as it was for instance observed in SiAlON glasses (in which bivalent oxygen atoms are partially substituted by trivalent nitrogen atoms) [336]. As a consequence of nitrogen incorporation within the silica network, an increase of the elastic moduli with 30% was observed upon replacing one out of five oxygen ions with nitrogen [337].

Studies on the high-temperature apparent shear viscosity of SiOC materials (from bending or compression creep experiments) indicate that at a specific temperature, the viscosity of SiOC glasses is several orders of magnitude higher than that of amorphous silica. This consequently indicates that the glass transition temperatures for SiOC glasses are significantly higher than that of vitreous silica. Thus, silicon oxycarbides have glass transition temperatures in the range of 1300 to 1350 °C, far beyond the glass transition temperature of vitreous silica (1190 °C). At temperatures exceeding 1350 °C, a creep hardening effect was observed and related to crystallization processes [333].

The unique high-temperature creep behavior of silicon oxycarbides was assumed to rely not only on the refractoriness of the carbon-containing glassy network Si*_x_*O*_y_*C*_z_*, but also on their nano/microstructure.

A recent study considers SiOC materials consisting of two continuous (and interpenetrating) phases, *i.e.*, silica and carbon. Thus, silica is considered to be continuous and “embedded” within a continuous carbon “skin”. This microstructure consideration was shown to be able to rationalize not only the creep rates of SiOC-based materials, but also the activation energies of creep, which were shown to be strongly affected by the nanodomain size of silica (*i.e.*, the “mesh size” of the carbon network within the microstructure of SiOC) [335]. In the studied temperature range (1000–1300 °C), the mechanism of creep was modeled with the Jeffreys viscoelastic model [338] and supported the proposed microstructural model in SiOC. Thus, two rheological contributions were identified: (i) a high viscous answer, coming from the silica rich network; and (ii) an elastic response from the segregated carbon phase within the samples. Moreover, two distinct effects of the carbon phase on the HT creep behavior of SiOC were identified: the effect of the carbon presence within the SiOC network (the “carbidic” carbon), which was shown to significantly increase the viscosity and in the same time to strongly decrease the activation energy for creep, as compared to vitreous silica; additionally, the segregated carbon phase (the “free” carbon) was shown to affect the viscosity and the activation energy of creep in SiOC and to the creep behavior in phase-separated silicon oxycarbides.

**Figure 16:**
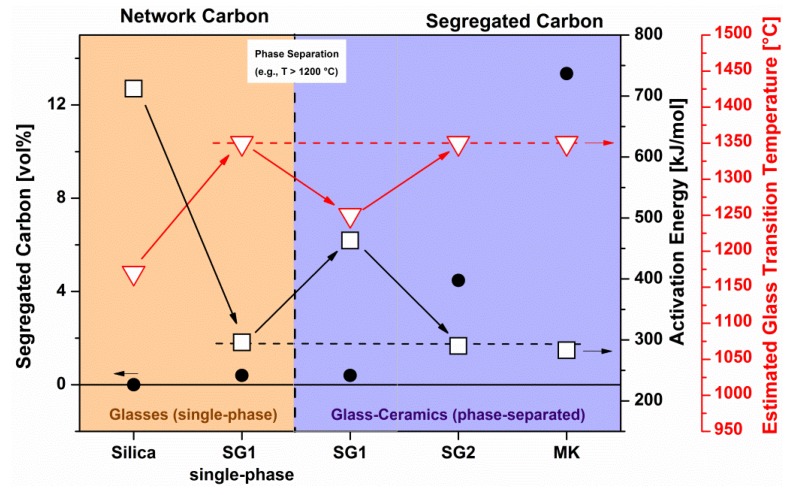
Dependence of the activation energy (red triangles) and *T*_g_ (black squares) on the content of segregated carbon (filled circles) in silicon oxycarbides (reprinted with permission from Wiley) [338].

From the HT creep study on SiOC materials it was concluded that single-phase SiOC glasses exhibit relatively large *T*_g_ values (1350–1400 °C) and rather low activation energies for creep (*ca.* 280–300 kJ/mol) and consequently they are materials of choice for near-zero-creep applications at HT (see Figure 14). However, they suffer upon long-term exposition to temperatures beyond 1000 °C from phase-separation processes. Phase-separated SiOC glasses were shown to have significantly lower *T*_g_ values and larger activation energy for creep than those of their single-phase counterparts. Their creep behavior can be significantly improved by incorporation of segregated carbon. Interestingly, small contents of segregated carbon are sufficient to lead to phase-separated SiOC samples with similar *T*_g_ and *E*_a_ values as compared to single-phase silicon oxycarbide (Figure 16). Thus, if high-temperature applications are anticipated (*i.e.*, above 1000 °C), SiOC compositions containing segregated carbon are mandatory in order to provide an improved creep resistance [338].

**Figure 17:**
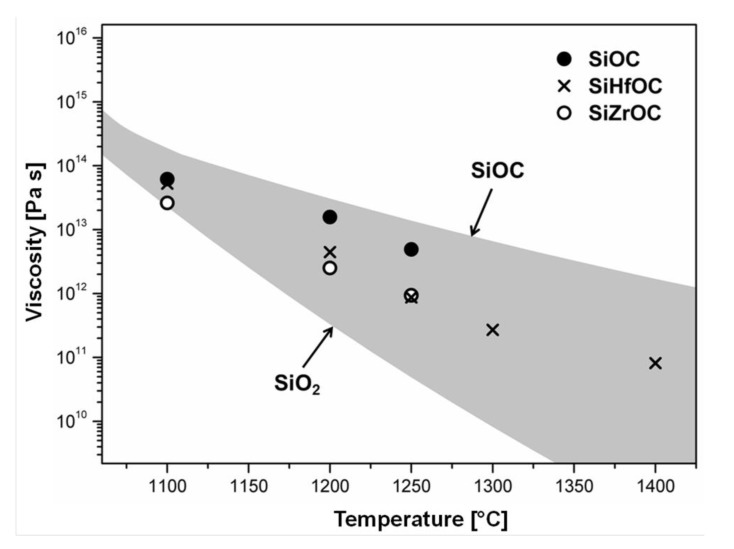
Viscosity of SiOC, SiZrOC, and SiHfOC as a function of temperature. The upper border of the gray region represent the temperature evolution of the viscosity of SiOC; whereas the lower border describes the evolution of the viscosity for vitreous silica (reprinted with permission from Wiley) [335].

It was shown that the modification of SiOC with additional elements (such as Al, Zr, Hf) induces an increase of the creep rates (*i.e.*, decrease of the shear viscosity). This was explained as a consequence of the strong decrease of the content of the segregated carbon phase [335]. Additionally, in the case of SiZrOC and SiHfOC, the modification of SiOC with Zr/Hf was demonstrated to lead to a significant increase of the activation energy for creep (from 286 kJ/mol for SiOC to 386 and 476 kJ/mol for SiZrOC and SiHfOC, respectively; see Figure 17) [335].

Silicon carbonitride ceramics (SiCN) were also studied with respect to their creep at high temperatures [339,340,341]. They show similar creep behavior and shear viscosity values as compared to those determined for SiOC materials. Also for SiCN a creep hardening behavior was observed and considered to rely on a nanoscale densification creep mechanism [340]. In comparison to SiOC and SiCN ceramics, SiBCN-based materials are much more refractory with respect to creep [341,342,343]. For SiBCN it was not possible to determine the glass transition temperature, as for instance the shear viscosity of SiBCN at 1450 °C (η ≈ 10^15^ Pa·s) was still significantly higher than the typical value observed for *T*_g_ (10^12^–10^12.6^ Pa·s). The *T*_g_ of SiBCN is expected to be *ca.* 1600 °C. Some case studies revealed that a thermal annealing of SiBCN prior to creep significantly improve their behavior [343]. However, the creep mechanisms in SiBCN (also generally in PDC-based nanocomposites though) are still poorly understood and have to be investigated in more detail.

### 5.2. Functional Properties

#### 5.2.1. Electrical Properties

Amorphous PDCs in the systems Si–O–C and Si–C–N exhibit electrical conductivities with values between those of semiconductors (e.g., SiC) and insulators (e.g., silicon nitride). The electrical properties of PDCs can be tuned by changing the composition of the preceramic polymers as well as by altering the annealing conditions [319]. Numerous studies related to the role of the free carbon phase present in PDC-NCs on their electrical properties were reported in the last two decades. According to most studies, the ceramic materials obtained upon pyrolysis at low temperatures (*i.e.*, *ca.* 1000 °C) are insulators; whereas high pyrolysis temperatures or high carbon content lead to SiOC-/SiCN-based materials having semiconducting behavior [173,185,344,345]. The addition of fillers can drastically affect the electrical properties of the PDC-NCs. For instance, the incorporation of molybdenum disilicide (MoSi_2_) into SiOC leads to an increase of the dc conductivity with up to 14 orders of magnitudes. For high disilicide contents the behavior of MoSi_2_/SiOC was found to be metallic, indicating the formation of MoSi_2_ conductive paths [345]. Also the electrical conductivity of SiCN was shown to increase with increasing pyrolysis/annealing temperature and with the content of segregated carbon [346,347]. AC impedance spectroscopy studies of SiCN/CNT nanocomposites showed that the addition of CNTs remarkably increases their conductivity as compared with that of pure SiCN. The conductivity of SiCN (*ca.* 10^−9^ S/cm) indicate that the SiCN matrix can be considered as an insulating material; whereas the addition of *ca.* 1 vol% multi-walled CNTs induced an increase of the conductivity with 5 orders of magnitude higher (10^−4^ S/cm), indicating a low percolation threshold [348].

Boron incorporation into the SiCN system leads to a significant increase of the conductivity. The room temperature conductivity of SiBCN was 4 orders of magnitude higher than that of SiCN. The SiBCN ceramics annealed at high temperatures exhibited *p*-type conductivity which was related to a compensation mechanism involving carrier generation from both nitrogen and boron [349,350].

**Figure 18:**
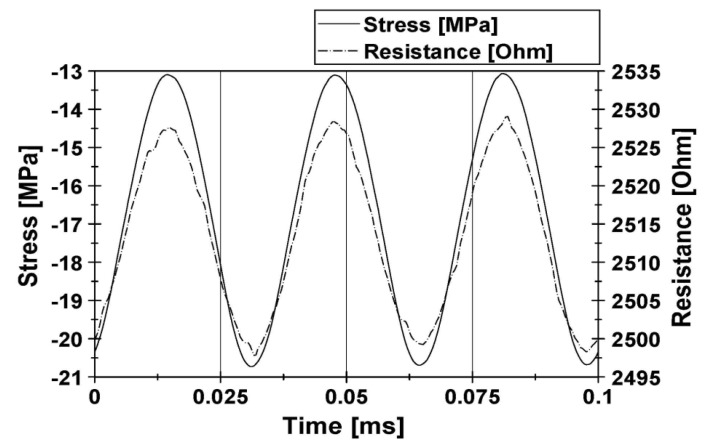
Piezoresistive response in silicon oxycarbide shown as the change of the electrical resistance upon applying a mechanical loading on the sample (reprinted with permission from Wiley) [353].

Recently it was shown that PDC-NCs exhibit unusually high piezoresistive coefficients (up to values of ~10^3^ as for SiCN-based materials [351]). The piezoresistive effect obeys the tunneling-percolation model [352] which is in good agreement with the formation of conductive graphene-like sheets within the PDC matrix. Also silicon oxycarbide-based PDC materials were found to show piezoresistive effect, with strain sensitivities (*k* factors) of *ca.* 145 (Figure 18) [353]. However, the piezoresistive effect in SiOC was observed only in ceramics annealed at a rather high temperatures (*i.e.*, 1400 °C), whereas the ceramics pyrolyzed at lower temperatures (1100–1300 °C) are not piezoresistive [353,354].

In two recent papers the high-temperature evolution of the piezoresistive effect in PDC-NCs was reported [355,356]. The determined values of the *k* factor for SiOC and SiOCN are significantly higher than those of well-ordered carbon and were found to decrease with increasing temperature. Rather high *k* values (k ~ 10^3^) have been reported for C/SiOCN in the temperature range 700 °C < *T* < 1000 °C [355]. Whereas the gauge factor of C/SiOC was determined in the range of *ca.* 10^2^ for temperatures from 1000 to *ca.* 1400 °C, its temperature dependence indicates a direct correlation with activated electronic transport (*E*_A_ ≥ 0.3 eV for *k*) [356]. The values of *k* at high temperatures for SiOC and SiOCN indicate their potential as robust materials for piezoresistive force and pressure sensing under extreme environments.

#### 5.2.2. Magnetic Properties

Several preparative methods for the synthesis of iron-containing PDC-NCs with interesting magnetic properties were reported. For instance, various approaches were reported in the last decades to synthesize iron silicide-based ceramic nanocomposites from tailored single-source precursors [357]. The synthetic principle involves the transformation (usually via heat treatment) of suitable single-source precursors into single-phase amorphous materials which subsequently phase separate and crystallize upon formation of ceramic (nano)composites containing iron silicides dispersed within their microstructure [154]. The single-source-precursor synthesis techniques have several advantages when considering the preparation of ceramic monoliths with tailor-made/tunable properties: By adjusting the molecular structure/architecture of the single-source precursor as well as its Fe and Si content and using appropriate pyrolysis conditions, ceramic composites with tunable magnetic properties can be prepared. For instance, the magnetic properties of SiFeC-based ceramic composites prepared upon ring-opening polymerization followed by cross-linking and pyrolysis of a [1]silaferrocenophanes were shown to be tunable between a superparamagnetic and a ferromagnetic state (see also Section 2.2 and Section 4.2.). This was demonstrated to depend on the size of the magnetic nanoprecipitations in the ceramic materials [120]. Additionally, the single-source-precursor technique allows for producing ceramic monoliths, coatings, fibers, *etc.* without additives at relatively moderate temperatures [6].

One convenient access to iron silicide-based ceramic nanocomposites involves the use of polymeric precursors which are modified with suitable iron-containing compounds prior to their conversion into the ceramics (see Table 1). In the case of polycarbosilane-derived ceramic composites, the pyrolysis of iron nitrate- [358] or acetylacetonate-modified precursors [359] leads to iron silicide-based ceramic composites, whereas an iron carbonyl-modified polycarbosilane was shown to convert into Fe/SiC ceramic nanocomposite [360].

Modification of a PSZ with Fe_3_O_4_ [361] or FeCl_3_ [362] followed by pyrolysis leads to Fe/SiCN nanocomposites, whereas the reaction of PSZ with Fe(CO)_5_ [215] or Fe powder [216] produces Fe_3_Si/SiCN composite materials upon pyrolysis. In an additional study, the ceramization of a ferrocene/hexamethyldisilazane-derived copolymer was shown to deliver Fe_3_C/Si_3_N_4_ composites [363].

**Table 1:** Selected examples of Si–Fe–C–X ceramics (X = O, N) derived from iron-modified preceramic polymers.

Ceramic material	Si-containing precursor	Fe-containing precursor	Pyrolysis conditions	Fe-containing crystalline phase(s)	References
Si–Fe–C–(O)	Polycarbosilane	Fe(NO_3_)_3_	1000–1200 °C (Ar)	Fe_3_Si	[358]
		Fe(acac)_3_	1300 °C (Ar)	Fe_5_Si_3_ (major), Fe_3_Si, FeC	[359]
		Fe(CO)_5_	1000 °C (N_2_)	Fe	[360]
Si–Fe–C–N–(O)	Polysilazane	Fe_3_O_4_	1000 °C (N_2_)	Fe (major), Fe_2_SiO_4_	[361]
		FeCl_3_/THF	1000 °C (N_2_)	Fe_3_Si	[362]
		Fe(CO)_5_	1100 °C (Ar)	Fe_3_Si	[215]
		Ferrocene	1200 °C (Ar)	Fe_3_C	[363]
		Fe (powder)	1100 °C (Ar)	Fe_3_Si	[216]
Si–Fe–O–C	Polysiloxane	FeCl_2_	1250 °C (N_2_)	Fe_3_Si	[364]
			1250–1300 °C (Ar)	Fe_3_Si, Fe_5_Si_3_	
		Fe(acac)_3_	1100 °C (Ar)	Fe_3_Si	[219,221]

Whereas several reports on the synthesis and magnetic properties of Fe/SiC(N) and Fe_3_Si/SiC(N) are known, there is not much information about the preparation and properties of ceramic composites consisting of crystalline iron silicide dispersed within a silicon oxycarbide matrix. Recently, the polymer-to-ceramic transformation of a FeCl_2_-modified polysiloxane was reported. Upon pyrolysis in nitrogen atmosphere the crystallization of Fe_3_Si along with Si_2_N_2_O and Si_3_N_4_ was found, whereas in argon atmosphere, Fe_3_Si, Fe_5_Si_3_ and β-SiC were formed [364]. Moreover, a study concerning the polymer-to-ceramic transformation of an iron-acetyl-acetonate-modified polysilsesquioxane indicates that probably the single-phase SiFeOC glassy materials obtained at moderate temperatures partitions into FeO and SiOC. As FeO is not stable under the strong carburizing conditions of the pyrolysis process, it converts *in situ* into Fe_3_Si. Interestingly, Fe_3_C was identified as intermediary phase prior to Fe_3_Si formation [219,221]. At temperatures above 1300 °C the amount of Fe_3_Si decreased and the crystallization of Fe_5_Si_3_ and β-SiC was observed. This behavior, which was observed also in the Si–Fe–C–N system, probably relies on the formation of a liquid Fe/Si/C alloy which, depending on the Fe:Si stoichiometry, induces either carbon segregation or SiC crystallization (SLS mechanism) [216]. In the case of SiFeOC and SiFeCN ceramics, the presence of a silicon-rich liquid alloy leads to the crystallization of silicon carbide and Fe_5_Si_3_ [216,219].

Also cobalt-containing PDC-NCs can be obtained from the complexations of the acetylene moieties of hyperbranched polyynes with cobalt carbonyls Co_2_(CO)_8_ and CpCo(CO)_2_ [365]. The inorganic-organic hybrid materials are thermally converted into nanostructured cobalt-containing ceramics. The resulting ceramics are highly magnetizable and show near-zero remanence and coercivity. Co-containing SiOC ceramics were prepared via cross-linking and pyrolysis of cobalt phthalate/polymethylphenylsiloxane blends [366]. Cobalt-modified SiCN materials were prepared from a cobalt carbonyl Co_2_(CO)_8_ modified polysilazane upon cross-linking and pyrolysis in inert atmosphere [215].

#### 5.2.3. Mechanical Properties

**Figure 19:**
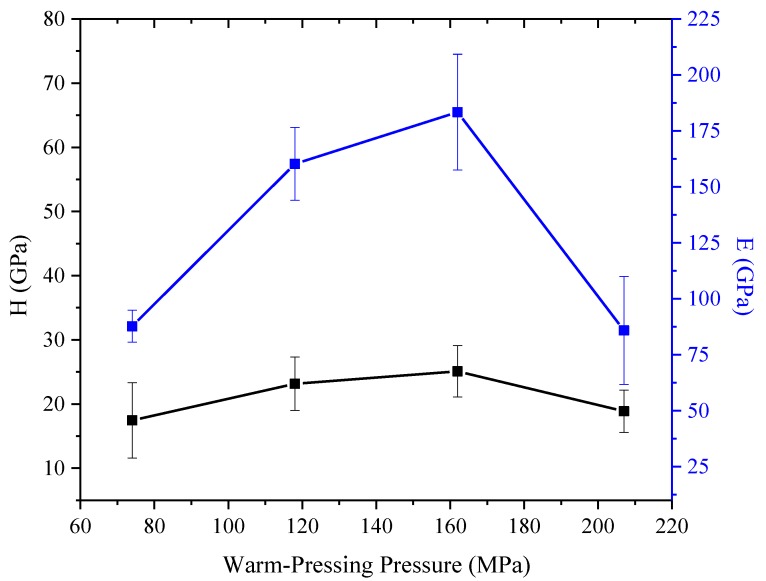
Evolution of the hardness and Young’s modulus of TiN/Si_3_N_4_ nanocomposites as functions of the applied warm-pressing pressure.

As previously described, the polymer-derived ceramic route provides a way to fabricate bulk silicon-based ceramics without the use of sintering additives either by plastic forming of preceramic polymers followed by pyrolysis [367,368,369] or by direct pyrolysis of polymers followed by spark plasma sintering process [370]. The room temperature mechanical properties, *i.e.*, hardness and elastic modulus, are key properties to proof the general applicability of these materials in particular in comparison to standard dense, sintered polycrystalline Si_3_N_4_ and silicon carbide (SiC). Numerous studies on the micro- and nanoindentation hardness of binary and ternary PDCs have been reported [332,371,372,373,374,375,376,377,378]. Nanoindentation test is one of the most effective and widely used methods to measure the mechanical properties of these materials. This technique uses the same principle as microindentation, but with much smaller probe and loads, so as to produce indentations from less than a hundred nanometers to a few micrometers in size. Galusek *et al.* [375] reported the hardness and elastic modulus of nearly dense cylindrical silicon carbonitride (SiCN) ceramics with a formal chemical composition of Si_1.00_C_0.67_N_0.80_ and bulk density of 2.32 g/cm^3^ produced by pyrolysis of a warm-pressed poly(hydridomethyl)silazane green body. It was reported that the mean hardness of the SiCN ceramics (13 ± 2 GPa) measured at a load of 250 mN was approximately half of the typical value of polycrystalline Si_3_N_4_ (24.9 ± 0.6 GPa) according to the presence of microscale defects and structural inhomogeneities (nanopores and clusters of free carbon within the ceramics). However, SiCN ceramics are harder and stiffer than SiO_2_ glass. SiCN ceramics display a similar behavior in mechanical properties than SiO_2_ glass most probably due to their global amorphous structure. Presumably because of the stronger SiOC and SiON covalent bonding, SiCN ceramics are both harder and stiffer than SiO_2_. Later, Cross *et al.* [379,380] studied the mechanical and tribological behavior of polyureamethylvinylsilazane-derived SiC*_x_*O*_y_*N*_z_* ceramics. Specimens in form of coupons were prepared by hot isostatic pressing up to different temperature from 800 to 1400 °C in nitrogen overpressure. All samples were X-ray amorphous and showed an increase of both the elastic modulus from ~100 to 184.0 GPa and the hardness from ~12 to 21.0 GPa with the increase of the pyrolysis temperature. Both values correlated strongly with the density of the specimens and the N/O ratio. More recently, nanoindentation technique was applied to polymer-derived ceramic TiN/Si_3_N_4_ [77]. Hardness values of 20.4 ± 1.7 GPa and elastic moduli of 171 ± 23 GPa have been measured on cylindrical specimens produced by warm-pressing at 110 °C of a titanium-containing polysilazane with a controlled Si:Ti ratio under different pressure followed by pyrolysis under ammonia up to 1000 °C and further heat-treatment under nitrogen up to 1300 °C [77]. However, since nanocomposites represent heterogeneous specimens in which very small nano-crystals grow, microindentation has been preferred. The Vickers hardness was found to reach 25.1 ± 4.0 GPa whereas a maximum Young’s modulus of 183.3 ± 25.9 GPa was measured. The values are closely related to the warm-pressing pressure which is applied during the plastic forming of titanium-containing polysilazanes (Figure 19): Both hardness and Young’s modulus gradually increase with the warm-pressing pressure from 74 to 162 MPa, then, mechanical properties decreased most probably due to the fact that the green sample has no open porosity, avoiding gaseous by-products to escape during pyrolysis generating cracks in the materials.

The high hardness of these nanocomposites is clearly related to the *in situ* controlled growth of the TiN nanophase in the amorphous Si_3_N_4_ matrix and the generation of very small TiN nano-crystals homogeneously distributed in the matrix.

SiBCN monoliths prepared by Spark Plasma Sintering of amorphous polymer-derived SiBCN powders under nitrogen exhibited Vickers hardness values which are closely related to the sintering temperature as well as the proportion of carbon in the powders [185]. Starting from Si_3.0_B_1.3_C_4.1_N_1.9_ powders, the mechanical properties of the derived monoliths increased from the sample sintered at 1500 °C to the sample sintered 1850 °C and remained almost constant in specimens sintered at 1850 and 1900 °C. A maximum Vickers hardness of 5.4 ± 1.2 GPa was measured whereas a maximum Young’s modulus of 102 ± 5 GPa was for samples sintered at 1900 °C. The modulus (and the hardness) is strongly correlated to the density of the specimens. The interesting point here is that the modulus increased linearly with the density of the sintered materials. Vickers hardness showed an increasing trend with the increase in processing temperature. This is mainly due to the lower residual porosity in the structure at higher SPS temperature resulting in the increase of the hardness. Furthermore, mechanical properties showed a significant variation when the raw powders exhibit lower carbon content. Hardness increased by to 15 ± 2 GPa and the Young’s modulus reached 150 ± 5 GPa for specimens obtained by SPS of Si_3.0_B_1.3_C_0.6_N_7.1_ powders at 1800 °C. This clearly shows that free carbon plays a prominent role with regard to the mechanical properties of polymer-derived (nano)composites. It could be concluded that ceramic closed to the “Si–B–N” composition (mainly composed of Si_3_N_4_ and BN) exhibit significantly higher hardness than those close to the “Si–B–C–N” system (composed of Si_3_N_4_ and SiC nanophases embedded in a turbostratic BN(C) matrix).

### 5.3. Applications of Ceramic Nanocomposites

#### 5.3.1. Ceramic Membranes

Microporous precursor-derived ceramic membranes for gas separation purposes were prepared in the recent past and they were shown to exhibit relatively high gas permeances and a good stability at high-temperatures which make them superior to polymer-based membranes concerning for instance dehydrogenation of hydrocarbons or steam reforming reaction for hydrogen synthesis [381]. Moreover, the single-source precursor route provides access to tailor-made micro-/meso-porous structure development [382].

Microporous membranes based on amorphous were prepared on permeable alumina porous supports having graded and layered porous structure [383,384]. The gas permeance for small molecules (such as He or H_2_) measured for amorphous silica-based membranes deposited on mesoporous anodic alumina capillary were significantly higher than those for bulkier gas molecules, indicating their potential in gas separation applications [385]. Amorphous silica-based membranes synthesized upon air pyrolysis of a polysilazane deposited on porous silicon nitride substrate revealed a hydrogen permeance of 1.3 × 10^−8^ mol·m^−2^·s^−1^·Pa^−1^ at 300 °C and a H_2_/N_2_ selectivity of 141, which is comparable with the permselectivity of other amorphous silica- or silicon oxycarbide-based membranes [386].

Also amorphous Si–C, Si–N, as well as Si–C–N and Si–B–C–N based ceramic membranes were prepared from appropriate single-source precursors. The possibility of using amorphous SiC ceramic membrane as a molecular sieve was firstly reported for a material prepared from polysilastyrene [387]. Moreover, SiC-based ceramic membranes were synthesized also upon thermal [384], e-beam [388] or chemical [389] curing of polycarbosilanes followed by pyrolysis in inert atmosphere. SiOC-based membranes with enhanced hydrogen permselective were prepared via curing of polycarbosilanes in air and subsequent pyrolysis in argon [390,391].

Amorphous silicon nitride-based ceramic membranes were prepared by ammonolysis of a polysilazane at 650 °C. The as-synthesized membrane showed a hydrogen permeance of 1.3 × 10^−8^ mol·m^−2^·s^−1^·Pa^−1^at 200 °C and a H_2_/N_2_ selectivity of 165, whereas after hydrothermal treatment at 300 °C the permeance was higher than 1.0 × 10^−7^ mol·m^−2^·s^−1^·Pa^−1^ at 300 °C with H_2_/N_2_ selectivity beyond 100 [382].

**Figure 20:**
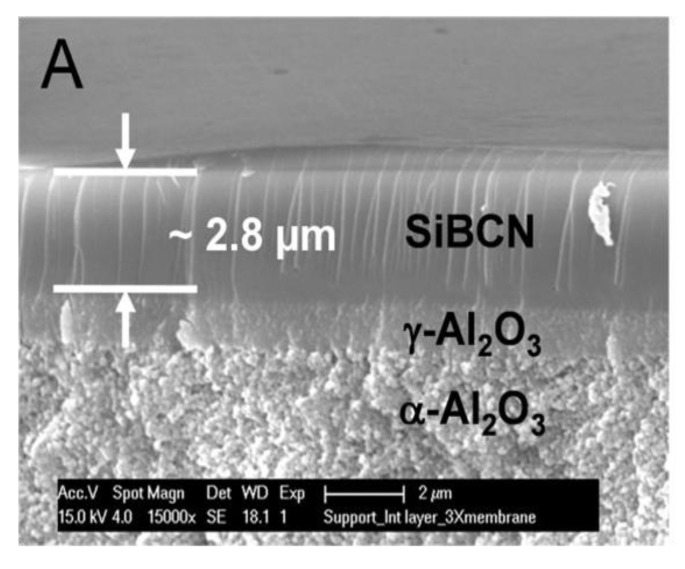
Cross section of a multilayered a-SiBCN/γ-Al_2_O_3_/α-Al_2_O_3_ membrane (reprinted with permission from reference [394]; Copyright 2010 Wiley).

Other preceramic polymers such as polysilazane, polyborosilazanes or polysilylcarbodiimides were used for the preparation of high-temperature stable amorphous ceramic membranes in the systems Si–C–N and Si–B–C–N. Mesoporous SiCN-based ceramic membranes (pore sizes in the range from 2 to 5 nm) were prepared via pyrolysis of polysilylcarbodiimide-based precursors which were synthesized from a non-oxidic sol–gel process based on reactions of bis(trimethylsilyl)carbodiimide with chlorosilanes [392]. Amorphous SiBCN-based ceramic membranes exhibiting a trimodal pore size distribution (0.6, 2.7 and 6 nm) were prepared on macroporous alumina supports via dip-coating and pyrolysis of a polyborosilazane [393]. Also, a multilayered a-SiBCN/γ-Al_2_O_3_/α-Al_2_O_3_ membrane with gradient porosity was prepared and investigated with respect gas separation behavior. A permeance of 1.05 × 10^−8^mol·m^−2^·s^−1^·Pa^−1^ and a H_2_/CO permselectivity of 10.5 were determined, showing its potential for applications such as hydrogen purification (Figure 20) [394].

In order to enhance the hydrothermal stability of the PDC-NCs gas separation membranes, single-source precursors based on metal-modified preceramic polymers were used, as for the case of Ni/SiCNO, WC(O)/SiOC or WN/SiOCN nanocomposites. The *in situ* precipitation of the metallic or ceramic nano-particles within the SiOC/SiOCN matrix was found to be responsible for the improved stability of the ceramic membranes in aggressive environments [395,396].

A recent systematic study reports on specific synthetic parameters which can be used in order to tune the chemical composition, (micro/meso)porosity and the specific surface area of PDC-NCs-based membranes. Thus, different preceramic polymers (e.g., polysiloxanes, polysilazanes) were shown to be able to develop stable microporosity and large specific surface area upon ceramization at moderate temperatures (600–700 °C) in ammonia atmosphere [397].

Due to their tunability as well as because of their robustness at high temperatures and in hostile environments, PDC-NC-based membranes might be the devices of choice for high-temperature gas separation applications.

#### 5.3.2. Porous Materials for Hydrogen Generation and Storage

Proton exchange membrane fuel cell-based (PEMFC) systems are attractive alternatives to current energy conversion technologies due to their potential to directly convert chemical energy such as hydrogen into electrical energy. They consist of three subsystems—fuel cell stack, hydrogen generator, and hybrid power management system. They display high efficiency, fast responses to loads and they have potentially zero emissions (except water). One of the most key challenges is the controlled release of hydrogen to meet the overall energy requirements for civil vehicle applications such as for drones. For this purpose, boron- and nitrogen-based chemical hydrides are attractive to be potential hydrogen carriers for PEM fuel cells owing to some very advantageous features: they can store and produce hydrogen “on-demand”, they are stable during long periods of storage without usage, they are non-flammable and non-toxic, they are hydrogen-rich compounds (large gravimetric energy densities) and side-products can be recyclable depending on the hydrides. Here we distinguish the liquid-phase hydrogen carriers such as the alkaline solution of sodium borohydride (NaBH_4_) to generate hydrogen by hydrolysis more suitable for portable applications and hydrogen storage materials such as ammonia borane (AB) which can release pure hydrogen at moderate temperatures by thermolysis for automotive applications.

Polymer-derived ceramics can be used in both types of applications as catalyst supports for hydrogen release from NaBH_4_ during its hydrolysis and as host material to confine AB with the objective to lower the temperature threshold for the H_2_ release and the emission of gaseous by-products such as ammonia and borazine.

Concerning the first aspect, PDCs find their interest according to the severe conditions of the reaction: hydrolysis is a strong exothermal heterogeneous reaction and the sodium borate which is formed is a strong base, *cf.* NaBH_4_ (aq) + 4 H_2_O → 4 H_2_ + NaB(OH)_4_ (aq) + Heat.

As soon as it is produced, the solution becomes basic. As a consequence, the application of MOFs, zeolites and metal oxides in this catalytic hydrolysis is somehow limited because their hydrothermal stability may turn to be poor in the severe conditions imposed by the reaction, leading in general to the collapse of the porous structure. To address the issue of highly stable supports to produce H_2_ from the alkaline solution of NaBH_4_, the use of ordered mesoporous polymer-derived SiAlCN powders as a support of Pt (which is required for an efficient hydrolysis reaction) allowed generating important volume of hydrogen after 2 h of reaction at 80 °C [247]. The performance was strongly correlated to the specific surface areas and pore volumes of the support: the highest specific surface area and pore volume are, the highest volume of hydrogen is generated. However, ordered mesoporous powders have necessary some difficulties in practical use, *i.e.*, in the scale of the demonstrator reactor. Within this context, further investigations concerning the preparation and application of monolithic PDCs and nanocomposites with tailored porosity are required.

Concerning the second aspect, nanoconfinement, appears to be one of the most efficient strategies to release pure hydrogen at low temperature for AB [398]. Gutowska *et al.* [399] showed that AB confined in the mesoporosity of silica SBA-15 has improved dehydrogenation behaviour in comparison to the pristine hydride, with an onset at 70 °C and the liberation of borazine-free H_2_. Since this pioneering work, various nano-scaffolds have been studied and reported: among others, carbonaceous hosts such as mesoporous CMK-3 [400] or nanotubes [401], polymethyl acrylate [402], magnesium metalorganic frameworks (MOF) [403,404], zeolite X [19] or silica hollow nanospheres [405].

The destabilization of AB is generally explained by two phenomena. The first one is the nanosizing of the hydride particles; it results in defect sites that initiate the dehydropolymerization of AB at lower temperatures. The second one is associated with H^δ+^···H^δ−^ surface interactions, with H^δ−^ of the BH_3_ moiety of AB and H^δ+^ belonging to surface/terminal hydroxyl groups (–O–H) generally found on carbonaceous or oxide nanoscaffolds. Such acid-base interactions enhance H_2_ release but usually lead to an unstable material in room conditions [406].

One of the strategies is therefore focused on the use of nanoscaffolds free of reactive surface groups to be safe in room conditions. Boron nitride (BN), which can be produced as nanostructured and porous materials from precursors [248,407,408,409,410], has been used to confine AB. In particular, BN nanopolyhedrons with hollow core and mesoporous shell structure with a BET specific surface area of 200.5 m^2^·g^−1^, a total pore volume of 0.287 cm^3^·g^−1^ and a narrow pore size distribution centred at 3.5 nm have been used as nanoscaffolds of AB in order to improve its dehydrogenation properties [411,412]. The as-formed BN NPHs-confined AB is able to liberate H_2_ at temperatures as low as 40 °C. Over the range 40–80 °C, the as-generated H_2_ is pure, the only traces of by-product (*i.e.*, NH_3_) being detected at >80 °C. Considering the effective regenerability of AB, the composite material represents a safe and practical hydrogen storage material which open the way to a very broad set of non-oxide materials including ceramics and nanocomposites using single-source molecules.

#### 5.3.3. Heterogeneous Catalysis

**Figure 21:**
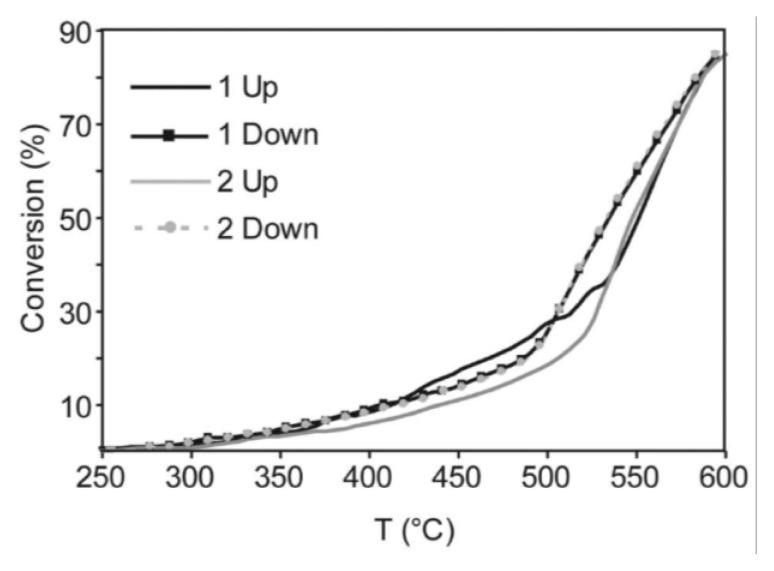
Methane conversion(activity) of highly porous and hierarchically ordered SiCN ceramics that integrate well-dispersed Pt nanoparticles as a function of the reaction temperature during two heating/cooling cycles (reprinted with permission from reference [151]; Copyright 2009 American Ceramic Society).

In Section 2.2, we presented the chemical modification of preceramic polymers using coordination compounds. In this approach, a metal transfer from the complex to the polymer chain can occur, giving rise to metal modified polymers, which are cross-linked and pyrolyzed, forming the metal-containing PDCs namely nanocomposites. Depending on the nature of the metal, as-obtained nanocomposites can be used for heterogeneous catalysis for reactions in harsh conditions because of the robust nature of non-oxide ceramic such as SiC and SiCN, the difference in terms of polarity and acidic properties in comparison to oxide ceramics, and their inertness and chemical resistance. As a proof-of-principle experiments, the catalytic activity and thermal stability of highly porous and hierarchically ordered SiCN ceramics that integrate well-dispersed Pt nanoparticles was tested in the total oxidation (combustion) of methane as shown in Figure 21 [151].

Current technologies for the conversion of natural gas to liquid products proceed by generation of carbon monoxide and hydrogen (syn-gas) that is then converted to higher products through Fischer-Tropsch chemistry. The direct, low-temperature, oxidative conversion of methane to an ester of methanol is best achieved with selected Pt complexes in high yield based on metal [413]. Platinum-supported SiCN ceramics showed the typical hysteresis behavior observed for platinum-catalyzed methane oxidation during heating and subsequent cooling cycles. The system combined excellent size control and high thermal stability of the catalytically active platinum with high structural flexibility rendering this system especially interesting for size-selective catalysis, monolith- and microreactor applications.

Interestingly, the use of coordination compounds with different metals allowed to generate a large range of metal-supported ceramic composition for various reactions. As an illustration, copper-containing silicon carbonitride ceramics (Cu@SiCN) by using silylaminopyridinato complexes showed catalytic activity towards the oxidation of cycloalkanes using air as oxidant [152]. As alkanes are inert and thermodynamically preferred total oxidation is highly competitive, there is a great need for catalysts that are able to convert simple alkanes into the corresponding mono-oxidation products. It was demonstrated that the selectivity issues can be addressed by tailoring the copper content as well as the nature of the metal loading in the polymer. Catalytic tests confirmed the applicability of these materials for the oxidation of cycloalkanes. Based on the same synthesis procedure, the same authors demonstrated that Ni@SiCN nanocatalysts were potential candidates for the hydrogenation of alkynes such as phenylacetylene into alkenes such as styrene [153]. For polymerization of styrene and obtain high-quality polymers, pure styrene feedstock is needed for a longer catalyst life. Even very small amount of phenylacetylene (which is used to produce styrene by dehydrogenation) in the styrene stream can deactivate the catalyst hence, styrene feed with very low concentration of phenylacetylene is mandatory. Monolithic microporous Ni@SiCN materials have been demonstrated as thermally robust until 500 °C in an oxidative environment and were selective hydrogenation catalysts for the conversion of phenylacetylene into styrene (selectivity ≥ 89% and conversion ≥ 99%). By changing the nature of the PDC phase (SiC), the resulting micro-, meso- and hierarchically porous Ni@SiC catalysts could be active and highly selective in the selective hydrogenolysis of the aromatic carbon-oxygen (C–O) bonds [155]. In particular, hierarchically structured Ni@SiC materials were the most active catalysts.

#### 5.3.4 Anode Materials for Secondary Li Ion Batteries

As described above (see Section 3), the ceramization of preceramic polymers delivers amorphous ceramics as intrinsically complex nano-structured materials composed of nano-domains of 1–3 nm in size, which persist up to very high temperatures [91,414,415,416]. An important feature of the PDC-NCs is their possibility to accommodate significant amounts of segregated carbon in their microstructure [80]. The nature, distribution and amount of the segregated carbon phase are correlated with the macromolecular architecture and decomposition behavior of the preceramic polymer. Within this context, carbon-rich SiCN and SiOC ceramics were shown to be suitable anode materials for Li-ion batteries [417,418,419,420].

The electrochemical properties of silicon oxycarbides were intensively studied in the last two decades. Especially materials consisting of large amounts of segregated carbon present within an amorphous SiOC matrix were identified as promising anode materials for secondary lithium ion battery (LIB) applications [421,422,423,424,425,426,427,428]. Within this context nanocomposites based on Si or Sn nanoparticles dispersed within SiOC were also studied [218,429].

Thus, in a case study, silicon oxycarbide/tin nanocomposites (SiOC/Sn) were prepared by chemical modification of two different polysilsesquioxanes with Tin(II) acetate and subsequent pyrolysis at 1000 °C. The obtained samples consisted of an amorphous SiOC matrix, *in situ* precipitated metallic Sn nanoparticles and segregated carbon. Galvanostatic cycling of both composites revealed superior cycling stability and rate capability of the C/SiOC/Sn nanocomposite containing large carbon content and was attributed to the soft, carbon-rich SiOC matrix, which was able to accommodate the volume changes related to the Li uptake and release of the Sn phase. The poor cycling stability found for the C/SiOC/Sn nanocomposite containing rather low amount of segregated carbon relates to mechanical failure of the stiff and fragile, carbon-poor matrix generated from it. Moreover, incremental capacity measurements emphasized that the two nanocomposites can incorporated different amounts of Li within the Sn phase. Thus, in the carbon-rich nanocomposite Li_7_Sn_2_ was formed; whereas the nanocomposite with lower carbon content exhibits the formation of Li_22_Sn_5_ was observed. The suppression of the formation of Li_22_Sn_5_ in the carbon-rich sample is rationalized by a restriction of the expansion of the matrix and thus prevention of a higher Li content in the Sn-Li alloy. For carbon-poor material, the matrix is severely degrading (fracture, cracking), providing an unlimited free volume for expansion and thus the formation of the lithium-rich Li_22_Sn_5_ phase is favored.

## 6. Conclusions

The use of tailor-made preceramic polymers provides a unique preparative access to ceramic nanocomposites possessing adjustable phase compositions and microstructures. Thus, a deep understanding of the interrelations between the features of the preceramic polymers and the structure and properties of the resulting ceramic nanocomposites represents a key parameter for a knowledge-based development of multifunctional materials based on polymer-derived ceramic nanocomposites.

However, despite numerous studies trying to elucidate these correlations, there is no systematic understanding yet available.

Consequently, following aspects should be intensively addressed in the future in order to provide a rational design of PDC-NCs with tailored compositions, microstructures and properties: (i) a straight-forward and knowledge-based synthetic access to preceramic single-source precursors with tailored chemical compositions and molecular architectures; (ii) a fundamental understanding of the ceramization process of the preceramic precursor to deliver PDC-NCs with designed compositions and microstructures; (iii) extensive experimental and modeling data are necessary in order to assess the properties of the PDC-NCs and to understand how they are affected/determined by the microstructural features of the nanocomposites.
